# Isolation of Stable Borepin Radicals and Anions

**DOI:** 10.1002/anie.202202516

**Published:** 2022-04-05

**Authors:** Kimberly K. Hollister, Wenlong Yang, Ranajit Mondol, Kelsie E. Wentz, Andrew Molino, Aishvaryadeep Kaur, Diane A. Dickie, Gernot Frenking, Sudip Pan, David J. D. Wilson, Robert J. Gilliard

**Affiliations:** ^1^ Department of Chemistry University of Virginia 409 McCormick Rd./PO Box 400319 Charlottesville VA 22904 USA; ^2^ Department of Chemistry and Physics La Trobe Institute for Molecular Science Latrobe University Melbourne 3086, Victoria Australia; ^3^ Fachbereich Chemie Philipps-Universität Marburg Hans-Meerwein-Strasse 4 35043 Marburg Germany

**Keywords:** Anions, Borepin, Boron, Carbenes, Radicals

## Abstract

Borepin, a 7‐membered boron‐containing heterocycle, has become an emerging molecular platform for the development of new materials and optoelectronics. While electron‐deficient borepins are well‐established, reduced electron‐rich species have remained elusive. Herein we report the first isolable, crystalline borepin radical (**2 a**, **2 b**) and anion (**3 a**, **3 b**) complexes, which have been synthesized by potassium graphite (KC_8_) reduction of cyclic(alkyl)(amino) carbene‐dibenzo[b,d]borepin precursors. Borepin radicals and anions have been characterized by EPR or NMR, elemental analysis, X‐ray crystallography, and cyclic voltammetry. In addition, the bonding features have been investigated computationally using density functional theory.

## Graphical abstract

The first examples of reduced borepins have been structurally characterized and offer new platforms for chemical reactivity.

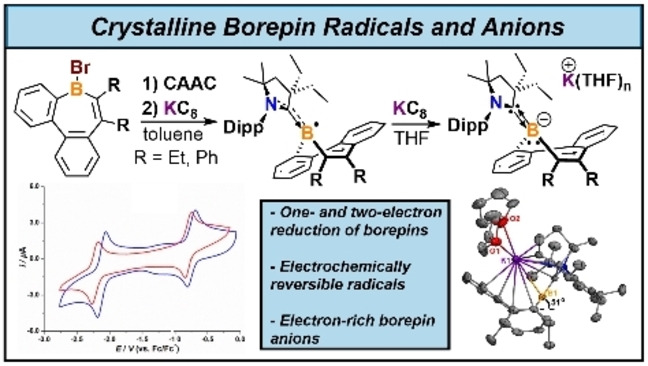

Boron‐doped polycyclic aromatic hydrocarbons (B‐PAHs) have been studied in a variety of different subfields of chemistry.^[1]^ Most notably, they have been used as a platform to elicit a wide range of optical and electronic properties in π‐conjugated materials.^[1]^ Borepin, a 6π‐electron B‐PAH, has recently received attention in materials chemistry,^[2]^ and as a means to understand fundamental issues of aromaticity in heterocyclic molecules.^[3]^ With few exceptions, nearly all structurally characterized tricoordinate borepins feature a neutral boron atom in a 7‐membered ring with three B−R single bonds (Figure 1A, R=C or N atom). Several neutral dibenzo‐^[4]^ and dithieno‐fused borepins[^3e^, ^5^] have been isolated and their optoelectronic properties have been investigated.[^2^, ^4^, ^5^] In 2019, we reported the molecular structures of the first examples of cationic borepins, borepinium ions (Figure 1B).^[6]^ Recently, fused diborepinium ions were synthesized and exhibited luminescent properties as a result of extended conjugation.^[7]^ Despite the ongoing interest in boron radicals^[8]^ and anions,^[9]^ the chemical synthesis and structural authentication of reduced, nucleophilic borepins are hitherto unknown (Figure 1C). This is likely due to their inherent high reactivity and the challenging nature of the experimental chemistry. Herein, we report the synthesis and isolation, X‐ray crystal structures, and theoretical studies of borepin radicals and anions—the first structurally characterized and storable examples of reduced borepin compounds. Unlike borepins in Figure 1A and B which are electron‐deficient, borepins in Figure 1C are electron‐rich, and the non‐planar conformation of the heterocycles result in a progressive concentration of electron density around boron and the formation of an exocyclic B=C double bond.

**Figure 1:**
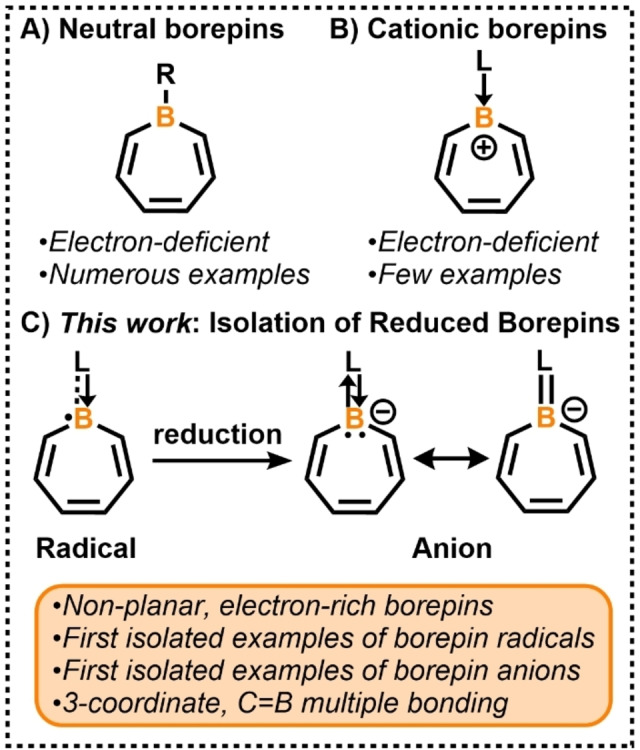
A) Neutral tricoordinate borepins. B) Electron‐deficient cationic borepins. C) First isolated examples of reduced borepins.

We began our studies by synthesizing dibenzo[b,f]borepin^[6]^ as a platform for reduction chemistry. However, numerous attempts at isolating radicals or anions proved challenging, and relevant compounds were prone to decomposition. Thus, the more sterically demanding neutral borepin **1 a** ^[10]^ was synthesized and allowed to react with 2,6‐(diisopropylphenyl)‐4,4‐diethyl‐2,2‐dimethyl‐pyrrolidin‐5‐ylidene (CAAC)^[11]^ in toluene to yield a carbene‐supported tetracoordinate borepin complex. However, after stirring at room temperature overnight, the colorless solution turned brown, and NMR analysis of an aliquot of the reaction mixture indicated that there was a substantial amount of **1 a** remaining. The difficulty in achieving complete conversion is likely due to the sterically hindered boron center. Despite these synthetic challenges, the CAAC‐stabilized diphenyl‐substituted‐dibenzo[b,d]borepin radical (**2 a**) was isolated as a yellow crystalline solid (Figure S1A) in 56 % yield via a one‐pot synthesis in which **1 a** and CAAC were combined with one equivalent of KC_8_ in toluene at room temperature (Scheme 1). Following a similar procedure, the CAAC‐stabilized diethyl‐substituted‐dibenzo[b,d]borepin radical (**2 b**) was isolated as an orange crystalline solid (Figure S1B) in 61 % yield. Both radicals were obtained as analytically pure solids, which are stable in the solid‐state for at least six months. The radicals are also stable in hexanes under inert atmosphere for at least three months.

**Scheme 1:**
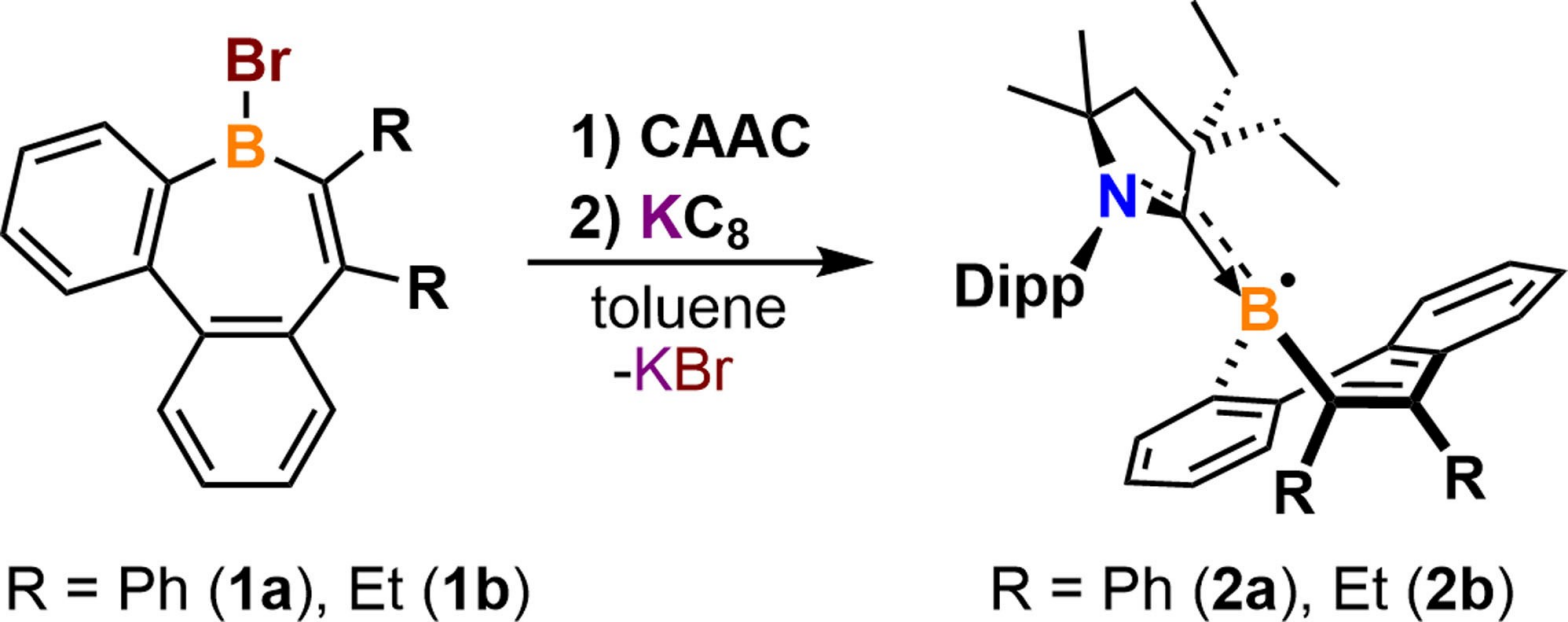
Syntheses of stable borepin radicals.

Air‐ and moisture‐sensitive single‐crystals of **2 a** and **2 b** suitable for X‐ray crystallographic analysis were obtained by recrystallization from concentrated hexanes solutions at −37 °C (Figure 2).^[12]^ For **2 a**, two chemically equivalent but crystallographically distinct molecules were observed in the unit cell (Figures 2 and S8). A planar geometry was observed for the tricoordinate boron center in both **2 a** and **2 b**, with the borepin rings adopting a boat‐shaped conformation. The boron centers lie 0.7576(51) Å above the plane containing C30−C23−C48−C43 in **2 a** and 0.7276(21) Å above the C28−C23−C36−C35 plane in **2 b**. The ^CAAC^C−B bond lengths in **2 a** [1.517(5) Å] and **2 b** [1.544(2) Å] are significantly shorter than that of the reported CAAC‐stabilized borepinium ion [1.637(8) Å].^[6]^ This is consistent with partial delocalization of the unpaired π‐electron onto the CAAC moiety in the radicals.

**Figure 2:**
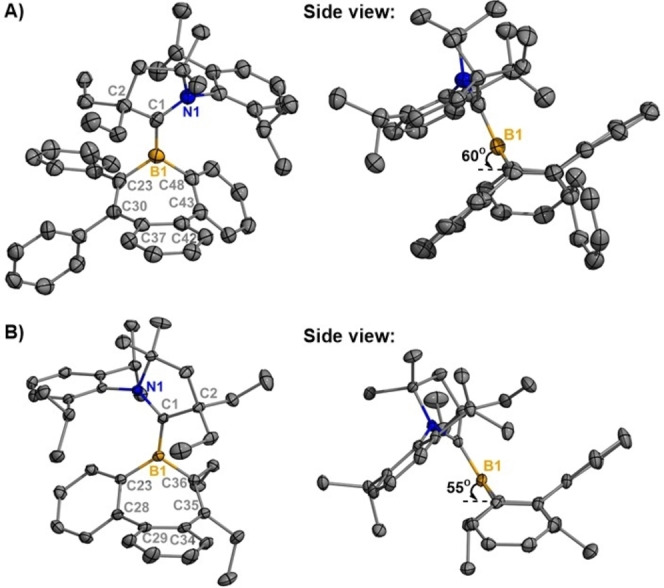
Molecular structures of **2 a** (A) and **2 b** (B) (thermal ellipsoids at 50 % probability; H atoms were omitted for clarity, for **2 a**: only one of two crystallographically independent molecules shown). Selected bond lengths [Å] and angles [°]: **2 a**: B1−C1 1.517(5), B1−C23 1.596(5), B1−C48 1.574(5); C1‐B1‐C23 122.7(3), C1‐B1‐C48 127.6(3), C23‐B1‐C48 109.2(3); **2 b**: B1−C1 1.544(2), B1−C23 1.591(2), B1−C36 1.594(2); C1‐B1‐C23 128.92(12), C1‐B1‐C36 121.48(12), C23‐B1‐C36 109.38(11).

The radical nature of compounds **2 a** and **2 b** was confirmed by EPR spectroscopy in toluene at room temperature, which displays different multiple‐line spectra centered at g=2.0019 (**2 a**) and 1.9993 (**2 b**) with complex hyperfine splitting (Figure 3). Simulations of the EPR spectra for **2 a** and **2 b** are well‐reproduced with a single spin system consisting of atoms N1, C1, and B1. Spin density plots reveal the unpaired electron in **2 a** and **2 b** is predominantly associated with the N1−C1−B1 π‐system (Figure 3). In complex **2 a**, the spin population is B1 (0.315, 31 %), C1 (0.421, 42 %), and N1 (0.245, 24 %), with minor contributions from the ligand and borepin heterocycle. In **2 b**, the unpaired spin distribution is similar to **2 a** [B1 (0.306, 30 %), C1 (0.437, 44 %), N1 (0.237, 24 %) (see ESI Table S3 for spin populations).

**Figure 3:**
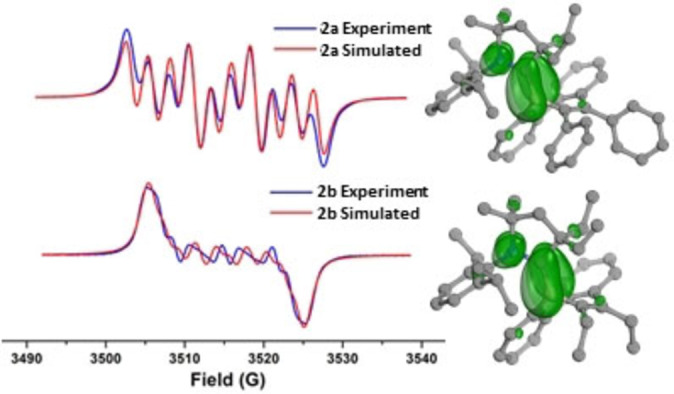
Continuous wave X‐band EPR spectrum and spin density plots of **2 a** (top) and **2 b** (bottom) in toluene solution at 298 K.

Cyclic voltammetry (CV) experiments were carried out in THF to investigate the redox properties of **2 a** and **2 b** (Figure 4). Both radicals give rise to two reversible waves. The diphenyl‐substituted dibenzo[b,d]borepin radical **2 a** exhibits slightly more positive reduction and oxidation potentials than the analogous diethyl‐substituted radical **2 b**. For the reduction of **2 a** and **2 b** to their corresponding anions, reduction potentials are observed at *E*_1/2_=−2.12 and −2.22 V, respectively (referenced against the ferrocene/ferrocenium (Fc/Fc^+^) redox couple). All cationic species are stable in THF with reversible oxidation waves at *E*_1/2_=−0.74 and −0.79 V for **2 a** and **2 b**, respectively. In contrast, the electrochemical features of neutral mesityl‐substituted dibenzo[b,f]borepin and mesityl‐substituted tetrabenzo[bc,ef]borepin^[4a]^ are significantly different, with quasi‐reversible reduction potentials at *E*_1/2_=−2.20 V and −2.56 V (THF, vs. Fc/Fc^+^), respectively, representing reduction to the corresponding radical anions. The reversible nature of borepin radicals **2 a** and **2 b** highlights the uniqueness of tricoordinate borepins stabilized by neutral carbene ligands. The UV/Vis absorption spectra of **2 a** and **2 b** in toluene reveals that both radicals exhibit strong absorption in the visible regions (Figure S2). TD‐DFT calculations (ωB97XD/def2‐SVP, SMD toluene) of UV/Vis absorbance agree with the experimental observations (Figure S13).

**Figure 4:**
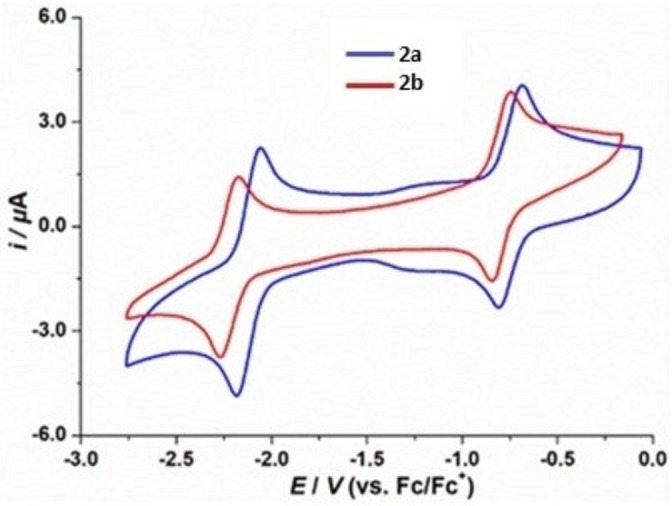
Cyclic voltammograms of **2 a** and **2 b** in THF/0.1 M [*n*Bu_4_N][PF_6_] at room temperature. Scan rate: 100 mV s^−1^.

Due to the appearance of a reversible reduction wave in the borepin radical CV experiments, we then sought to synthesize borepin anions by subsequent one‐electron chemical reduction. The CAAC‐stabilized diphenyl‐substituted dibenzo[b,d]borepin anion (**3 a**) was obtained as dark red crystals (Figure S1C) in 87 % yield after stirring a mixture of **2 a** with 2 equivalents of KC_8_ in THF at room temperature and recrystallizing from a THF/hexanes mixture at −37 °C (Scheme 2). The use of excess reducing agent resulted in cleaner conversion to **3 a**. Attempts to obtain NMR data on multiple batches of **3 a** proved to be challenging, with inseparable trace impurities of **2 a** resulting in a paramagnetic NMR in the majority of reaction trials.^[13]^ However, in two instances, sufficiently resolved ^1^H and ^11^B{^1^H} NMR spectra could be obtained and the solid was analytically pure by elemental analysis. The ^11^B{^1^H} resonance at 21.7 ppm is significantly upfield compared to the neutral borepins bearing tricoordinate boron centers (*δ* 48.0–68.0 ppm),^[2a]^ and can be attributed to a negatively charged, electron‐rich boron center. Following a similar synthetic procedure, the CAAC‐stabilized diethyl‐substituted dibenzo[b,d]borepin anion (**3 b**) was obtained as a red/orange solid (Figure S1D) in 71 % yield by reacting **2 b** with 1.1 equivalents of KC_8_. Compound **3 b** was fully characterized by NMR spectroscopy and elemental analysis. The ^1^H NMR spectrum in C_6_D_6_ shows a septet resonance at 4.10 ppm for the methine proton of the CAAC Dipp group, which is downfield compared to the free CAAC (3.18 ppm). The ^11^B{^1^H} NMR resonance at 22.4 ppm is consistent with the electron‐rich tricoordinate boron signal observed for **3 a**.

**Scheme 2:**
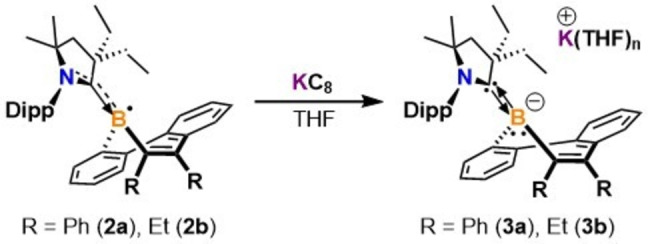
Syntheses of borepin anions.

Single crystals of **3 a** and **3 b** were obtained by recrystallization from a THF/hexanes mixture at −37 °C. Crystallographic analyses of **3 a** and **3 b** reveal that both molecules possess a trigonal planar geometry around the boron centers (Figure 5). No contacts are observed between the borepin core and potassium cation in **3 a**, whereas the cation in **3 b** has contacts with both the borepin and CAAC ligand. Similar to radicals **2 a** and **2 b**, the borepin rings in **3 a** and **3 b** adopt a boat‐shaped conformation where the boron centers lie 0.7690 Å above the plane containing C30−C23−C48−C43 (**3 a**) and 0.7531 Å above the plane containing C28−C23−C35−C38 (**3 b**). Significant shortening of the ^CAAC^C−B bond is observed upon reduction of **2 a** [1.517(5) Å] and **2 b** [1.544(2) Å] to their anions **3 a** [1.461(5) Å] and **3 b** [1.465(4) Å]. This observation is consistent with the formation of an exocyclic B=C double bond, similar to the bonding observed in methyleneboranes.^[14]^

**Figure 5:**
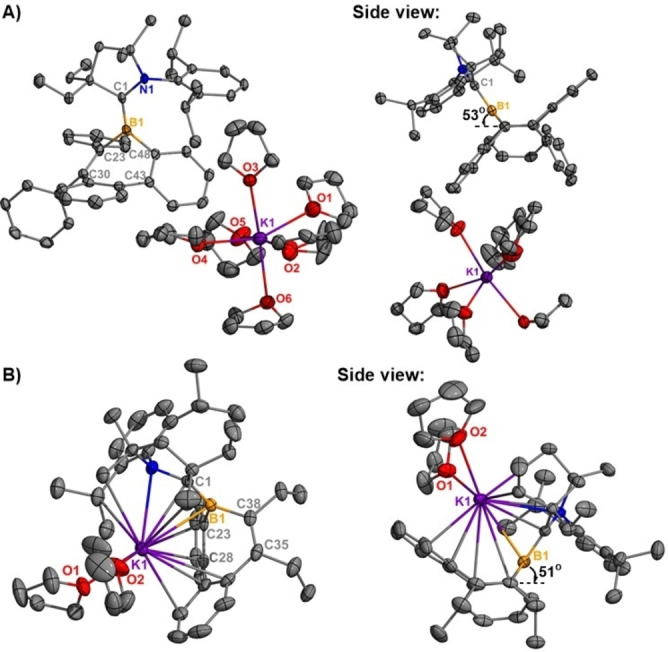
Molecular structures of **3 a** (A) and **3 b** (B) (thermal ellipsoids at 30 % probability, respectively; H atoms were omitted for clarity, one uncoordinated THF omitted for clarity (**3 a**), Selected bond lengths [Å] and angles [°]: **3 a**: B1−C1 1.461(5), B1−C23 1.624(5), B1−C48 1.597(5); C1‐B1‐C23 121.9(3), C1‐B1‐C48 129.6(3), C23‐B1‐C48 106.2(3); **3 b**: B1−C1 1.465(4), B1−C23 1.610(4), B1−C38 1.614(4), C1‐B1‐C23 129.3(2), C1‐B1‐C38 124.2(2), C23‐B1‐C38 106.54(19).

Theoretical calculations were performed to probe the electronic structure and bonding of compounds **2 a**, **2 b**, **3 a** and **3 b**. In addition, radical and anionic CAAC‐stabilized unsubstituted borepins were also examined to assess the conformational effects of borepin. Both radical and anionic unsubstituted borepins exhibit planar geometries with highly localized borepin π contributions to the HOMO (Figure S19). The deviation from planarity in substituted borepins is due to low‐frequency vibrations of the annulated rings and Et/Ph substituents. Although anionic borepins **3 a** and **3 b** are formally anti‐aromatic, the sizable displacement from planarity results in reduced π‐character of the borepin moiety and increased B1–C1 π‐character. Therefore, compounds **3 a** and **3 b** are best described as non‐aromatic systems. The HOMO of the anionic compounds display significant B1−C1 π‐bonding character with no contributions from the π‐system of the borepin ring (Figures S16 and S17). Hirshfield‐CM5 atomic charges were computed to confirm the radical and anionic character of the reduced borepins. Negative charges are assigned to the boron atoms in **2 a** (−0.167 *e*) and **2 b** (−0.171 *e*), with an increased negative charge in **3 a** (−0.296 *e*) and **3 b** (−0.297 *e*), consistent with one‐ and two‐electron reductions. The carbene carbon is positively charged in singly‐reduced complexes (**2 a** +0.129 *e*, **2 b** +0.125 *e*) while almost neutral in doubly‐reduced species **3 a** (+0.028 *e*) and **3 b** (+0.022 *e*), highlighting the polarization towards boron in anionic borepin.

Energy decomposition analysis in combination with natural orbitals for chemical valence (EDA‐NOCV)^[15]^ and calculations of Wiberg bond indices (WBI) were performed to shed light on the nature of bonding in **2 a** and **2 b** and their anionic analogues. WBI values for B1−C1 indicate that both radical (WBI=1.342 (**2 a**), 1.340 (**2 b**)) and anionic (WBI=1.533 (**3 a**), 1.525 (**3 b**)) compounds possess electron populations associated with delocalized bonds. In order to reflect the most suitable bonding situation in these complexes, we considered CAAC and borepin with different charges and electronic states (Tables S4–S7). In cases where more than one partitioning scheme is available, the size of the orbital interaction, Δ*E*_orb_, can be used as a probe to understand which one best describes the bonding situation.^[16]^ In **2 a** and **2 b**, doublet borepin radical binding with singlet CAAC through a donor–acceptor interaction was the most suitable bonding description. In **3 a** and **3 b**, doublet borepin radical binding with doublet anionic [CAAC]^−^ through an electron‐sharing π bond and dative σ bond was a marginally preferred bonding description compared to singlet borepin anion interacting with CAAC through donation/back‐donation. However, the small difference in Δ*E*_orb_ (ΔΔ*E*_orb_≈2.7 kcal mol^−1^) for the two models indicates that either description could reasonably be employed. The detailed numerical results of EDA‐NOCV for the preferred interacting scheme for radical and anionic complexes are provided in Table S8. The intrinsic interaction (Δ*E*_int_) between CAAC and borepin is quite strong in the anionic system (Δ*E*_int_ (kcal mol^−1^)=−202.6 (**3 a**), −195.5 (**3 b**)) as two electrons are involved in the π bond in contrast to the single electron in the neutral radicals (Δ*E*_int_ (kcal mol^−1^)=−155.1 (**2 a**), −161.1 (**2 b**)). The bonds between CAAC and borepin are slightly more covalent (Δ*E*_orb_≈49–52 %) than electrostatic (Δ*E*_elstat_≈42–44 %) in nature. Dispersion interaction accounts for 5–7 % of the total attraction. The breakdown of the Δ*E*_orb_ into pairwise orbital interactions shows that the strongest orbital interaction, Δ*E*_orb(1)_, originates from [borepin]←[CAAC] σ donation (51–60 % of total Δ*E*_orb_), whereas the next most important orbital term, Δ*E*_orb(2)_, is due to [borepin]→[CAAC] π backdonation for the neutral system, and [borepin]—[CAAC]^−^ electron‐sharing π bond formation for anionic complexes, which account for 23–33 % of total Δ*E*_orb_.

In conclusion, the first isolated examples of reduced borepin compounds have been synthesized as crystalline radicals and anions. The combined experimental and simulated EPR spectroscopic data for the radicals indicate that the chemical environment of the unpaired electron can be tuned based on the nature of the borepin substituents. Synthesis and isolation of stable organic radicals such as these species have potential applications in energy storage and conversion devices (e.g., organic batteries, photovoltaic devices, thermoelectric systems, optoelectronics).^[17]^ Redox‐flexible radicals are particularly interesting because of their ambipolar behavior—the ability to be selectively oxidized to closed shell cations or reduced to the respective anions—an important prerequisite for many energy‐relevant applications.^[17a]^ Reduction to the borepin anions not only serves as a key fundamental discovery, but these molecules could serve as chemical synthons for new types of materials with unusual bonding. Studies of this variety are currently underway in our laboratory and will be reported in due course.

## Conflict of interest

The authors declare no conflict of interest.

## Supporting information

As a service to our authors and readers, this journal provides supporting information supplied by the authors. Such materials are peer reviewed and may be re‐organized for online delivery, but are not copy‐edited or typeset. Technical support issues arising from supporting information (other than missing files) should be addressed to the authors.

Supporting Information

Supporting Information

## Data Availability

The data that support the findings of this study are available in the Supporting Information of this article.
