# Supplementary material for: Isolation of Stable Borepin Radicals and Anions
Source: Angew Chem Int Ed Engl. 2022 Apr 5;61(23):e202202516. doi: 10.1002/anie.202202516 (PMC9324096; doi:10.1002/anie.202202516)
Supplement: Supplementary file 2 — Supporting Information [file ANIE-61-0-s001.pdf]

Angewandte Chemie

Eine Zeitschrift der Gesellschaft Deutscher Chemiker

Supporting Information

Isolation of Stable Borepin Radicals and Anions

K. K. Hollister, W. Yang, R. Mondol, K. E. Wentz, A. Molino, A. Kaur, D. A. Dickie, G. Frenking, S. Pan, D. J. D. Wilson*, R. J. Gilliard, Jr.**

Supporting Information

Table of Contents

page

General Procedures……………………………………………………………………………………..2
Experimental Procedures……………………………………………………………………………..3-4
EPR Spectroscopy………………………………………………………………………………………6
UV-Vis Spectroscopy……………………………………………………………………………………6
NMR Spectral Data……………………………………………………………………………………..7-11
X-ray Data Collection Details………………………………………………………………………...12-15
Theoretical Calculations………………………………………………………………………………16-32
References………………………………………………………………………………………………33

General Procedures

All manipulations were carried out under an atmosphere of purified argon using standard Schlenk techniques or an MBRAUN LABmaster glovebox equipped with a –37 °C freezer. All reaction solvents were distilled over sodium/benzophenone. Deuterated solvents were purchased from Acros Organics and Cambridge and distilled over sodium/benzophenone prior to use. THF-d_8 was stored over Na/K alloy and filtered over freshly cut Na 1 hour prior to use. Glassware was oven-dried at 190 °C overnight. The NMR spectra were recorded at 298.15 K on a Varian 600 MHz and Bruker 600 MHz spectrometer. Proton and carbon chemical shifts are reported in ppm and are referenced using the residual proton and carbon signals of the deuterated solvent (^{1}H; C_6D_6, δ 7.16 ppm; ^{13}C; C_6D_6, δ 128.06 ppm). Abbreviations are as follows; s = singlet, d = doublet, t = triplet, p = pentet, hept = heptet, dd = doublet of doublets, td = triplet of doublets, m = multiplet. All boron signals were referenced to an external standard, $BF_3{\cdot}Et_2O$ (^{11}B: δ=0.00 ppm). EPR spectra were recorded using a Bruker X-Band EMX spectrometer (Bruker Biospin, Billerica, MA) equipped with an ER 4123D dielectric resonator. All EPR spectra were recorded using a 100 G magnetic field sweep, 1 G modulation, and 2.0-milliwatt incident microwave power at a temperature of 298 K. Single crystal X-ray diffraction data for **3b** were collected on a Bruker D8 Venture Photon III Kappa four-circle diffractometer system equipped with an Incoatec IμS 3.0 micro-focus sealed X-ray tube (Cu Kα, λ = 1.54178 Å) and a HELIOS MX double bounce multilayer mirror monochromator. All other crystallographic measurements were made on a Bruker Kappa APEXII Duo system equipped with a fine-focus sealed tube (Mo Kα, λ = 0.71073 Å) and graphite monochromator (**2b** and **3a**) or an Incoatec Microfocus IμS (Cu K_α, λ = 1.54178 Å) and multi-layer mirror monochromator (**2a, 3a**$^{\mathbf{Et_2O}}$ and **3b**$^{\mathbf{Et_2O}}$). Elemental analyses were performed at the University of Virginia Department of Chemistry using a Perkin Elmer 2400 Series II Instrument. UV-Vis absorption spectra were recorded using a Cary 60 UV-Vis Spectrophotometer. Electrochemical measurements were collected using a Pine WaveNow Potentiostat at a scan rate of 100 mV/s. The electrochemical cell consisted of a glassy carbon working electrode, Pt wire counter electrode and Ag/AgCl reference electrode. The supporting electrolyte was $[Bu_4N]^+[PF_6]^-$ and all potentials are reported relative to the ferrocene/ferrocenium couple. IPr[1] and CAAC[2] were prepared according to published procedures. Diphenylacetylene and diethylacetylene were purchased from Sigma-Aldrich and used as received.

Experimental Procedures

Synthesis of compound **2a** [$C_{48}H_{53}BN$]: To a 100 mL round bottom Schlenk flask containing a toluene (15 mL) solution of **1a** (42 mg, 0.10 mmol) and ^{Et}CAAC (31 mg, 0.10 mmol), KC_8 (14 mg, 0.10 mmol) was slowly added at room temperature. The reaction mixture gradually turned orange with stirring and after 14 hours the reaction was stopped. The resulting mixture was filtered to give an orange toluene solution. Toluene was removed under vacuum and the solid was extracted with hexanes (20 mL). The hexanes solution was concentrated to 5 mL, and crystallization at –37 °C afforded 37 mg of a yellow crystalline solid (56% yield). Anal. Calcd for $C_{48}H_{53}BN$: C, 87.60; H, 8.44; N, 2.22%. Found: C, 87.48; H, 8.33; N, 2.10%.

Synthesis of compound **2b** [$C_{40}H_{53}BN$]: To a 100 mL round bottom Schlenk flask containing a toluene (15 mL) solution of **1b** (33 mg, 0.10 mmol) and ^{Et}CAAC (31 mg, 0.10 mmol), KC_8 (14 mg, 0.10 mmol) was slowly added at room temperature. The reaction mixture gradually turned red with stirring and after 14 hours the reaction was stopped. The resulting mixture was filtered to give a red/orange toluene solution. Toluene was removed under vacuum and the solid residue was extracted with hexanes (20 mL). The hexanes solution was concentrated to 5 mL and crystallization at –37 °C afforded 34 mg of an orange crystalline solid (61%). Anal. Calcd for $C_{40}H_{53}BN$: C, 86.00; H, 9.56; N, 2.51%. Found: C, 85.80; H, 9.81; N, 2.31%.

Synthesis of compound **3a** [$C_{48}H_{53}BKN \cdot 6THF$]: To a solution of **2a** (41 mg, 0.063 mmol) in THF (3 mL), KC_8 (17 mg, 0.12 mmol) was added in one portion and the bright red solution immediately darkened. The reaction was stirred for 17 hours and filtered to afford a deep red solution. The solution was concentrated and an equal ratio of hexanes was added. Dark red crystals were grown at -37 °C (62 mg, 0.055 mmol, 87%). ^{1}H NMR (600 MHz, THF-d_8, ppm) δ 7.23 – 7.20 (m, 1H, Ar*H*), 7.17 – 7.14 (m, 1H, Ar*H*), 7.11 – 7.08 (m, 2H, Ar*H*), 7.00 (dd, *J* = 7.4, 1.4 Hz, 1H, Ar*H*), 6.93 – 6.89 (m, 2H, Ar*H*), 6.87 – 6.80 (m, 5H, Ar*H*), 6.77 – 6.70 (m, 2H, Ar*H*), 6.63 – 6.57 (m, 2H, Ar*H*), 6.48 (m, 3H, Ar*H*), 6.33 (td, *J* = 7.3, 1.4 Hz, 1H, Ar*H*), 6.03 (dd, *J* = 7.5, 1.5 Hz, 1H, Ar*H*), 3.73 (p, *J* = 6.8 Hz, 1H, C*H*(CH_3)$_2$), 3.01 (p, *J* = 6.7 Hz, 1H, C*H*(CH_3)$_2$), 1.97 (dd, *J* = 14.8, 7.5 Hz, 1H, C*H*$_2$$CH_3$), 1.82 – 1.76 (m, 1H, C*H*$_2$$CH_3$), 1.67 – 1.62 (m, 1H, C*H*$_2$$CH_3$), 1.60 (d, *J* = 11.9 Hz, 1H), 1.54 (dd, *J* = 15.0, 7.6 Hz, 1H, C*H*$_2$$CH_3$), 1.46 (d, *J* = 11.7 Hz, 1H), 1.34 – 1.25 (m, 4H), 1.18 (d, *J* = 6.6 Hz, 3H), 1.04 (s, 3H, C*H*$_3$), 1.00 (d, *J* = 7.0 Hz, 3H), 0.89 (dd, *J* = 7.0, 2.0 Hz, 6H), 0.75 (t, *J* = 7.6 Hz, 3H, CH_2C*H*$_3$), 0.68 (s, 3H, C*H*$_3$), 0.62 (t, *J* = 7.5 Hz, 3H, CH_2C*H*$_3$), 0.03 (d, *J* = 6.7 Hz, 3H, CH(C*H*$_3$)$_2$). ^{11}B NMR (192 MHz, THF-d_8, ppm) δ 21.74. Anal. Calcd for $C_{48}H_{53}BKN \cdot (THF)_6$: C, 76.77; H, 9.04; N, 1.24%. Found: C, 76.74; H, 9.10; N, 1.59%. *Note: Despite numerous reaction trials sufficiently resolved and clean ^{1}H and ^{11}B NMR spectra were only obtained in two instances, and a sufficiently resolved ^{13}C NMR spectrum could not be obtained. The crystalline bulk product (Figure S1C) is prone to contamination by an ultra small amount of borepin radical **2a**, which was confirmed by EPR spectroscopy. However, the sample meets standard criteria

for purity as determined by elemental analysis. Our speculation is that a trace impurity in the deuterated solvent results in reversion to **2a**, rather than incomplete reduction during the reaction. Addition of excess KC_8 or alkali metals to the reaction yields the same result by NMR.

Synthesis of compound **3b** [$C_{40}H_{53}BKN·2THF$]: To a solution of **2b** (74 mg, 0.13 mmol) in THF (3 mL), KC_8 (20 mg, 0.14 mmol) was added in one portion and the bright red solution immediately darkened. The reaction was stirred for 17 hours and filtered to afford a deep red solution. The THF was removed under vacuum to isolate a red/brown solid (70 mg, 0.094 mmol, 71%). ^{1}H NMR (600 MHz, C_6D_6, ppm) δ 7.65 (d, *J* = 7.8 Hz, 1H, Ar*H*), 7.40 (d, *J* = 7.5 Hz, 1H, Ar*H*), 7.36 (d, *J* = 7.5 Hz, 1H, Ar*H*), 7.23 (t, *J* = 7.5 Hz, 1H, Ar*H*), 7.14 (d, *J* = 8.0 Hz, 1H, Ar*H*), 7.09 (t, *J* = 7.5 Hz, 1H, Ar*H*), 6.91 (t, *J* = 7.5 Hz, 1H, Ar*H*), 6.87 (t, *J* = 7.4 Hz, 1H, Ar*H*), 6.78 (t, *J* = 7.3 Hz, 1H, Ar*H*), 6.76 (d, *J* = 7.7 Hz, 1H, Ar*H*), 6.17 (d, *J* = 7.6 Hz, 1H, Ar*H*), 4.10 (hept, *J* = 6.6 Hz, 1H, C*H*(CH_3)$_2$), 3.51 (m, 4H, THF), 3.02 (m, 1H, C$H_2$$CH_3$), 2.87 (m, 1H, C$H_2$$CH_3$), 2.69 (m, 1H, C$H_2$$CH_3$), 2.52 (m, 1H, C$H_2$$CH_3$), 2.45 (m, 1H, C$H_2$$CH_3$), 2.35 (m, 1H, C$H_2$$CH_3$), 2.24 (m, 1H, C*H*($CH_3$)$_2$), 2.11 (d, *J* = 12.6 Hz, 1H, CH_2), 1.82 (d, *J* = 6.3 Hz, 3H, CH(CH_3)$_2$), 1.79 (m, 1H, C$H_2$$CH_3$), 1.74 (d, *J* = 12.9 Hz, 1H, CH_2), 1.59 (m, 1H, CH_2CH_3), 1.46 (d, *J* = 6.6 Hz, 3H, CH(CH_3)$_2$), 1.40 (m, 4H, THF), 1.25 (t, *J* = 7.9 Hz, 6H, CH_2CH_3), 1.09 (m, 3H, CH_2CH_3), 1.01 (d, *J* = 6.7 Hz, 3H, CH(CH_3)$_2$), 0.94 – 0.88 (m, 3H, CH_2CH_3), 0.84 (s, 3H, CH_3), 0.72 (s, 3H, CH_3), -0.10 (d, *J* = 7.1 Hz, 3H, CH(CH_3)$_2$).^{13}C NMR (201 MHz, C_6D_6) δ 150.35, 150.10, 148.35, 146.32, 145.10, 140.25, 133.97, 132.18, 129.08, 128.42, 126.96, 125.81, 125.43, 125.09, 124.18, 123.40, 123.10, 62.23, 51.45, 50.90, 39.29, 36.37, 33.45, 32.39, 29.73, 27.98, 27.55, 26.27, 25.69, 24.99, 24.70, 21.16, 16.83, 14.92, 12.69, 11.86. ^{11}B NMR (192 MHz, C_6D_6, ppm) δ 22.32. Anal. Calcd for $C_{40}H_{53}BKN·(THF)_2$: C, 77.70; H, 9.37; N, 1.89%. Found: C, 77.93; H, 9.59; N, 2.06%.

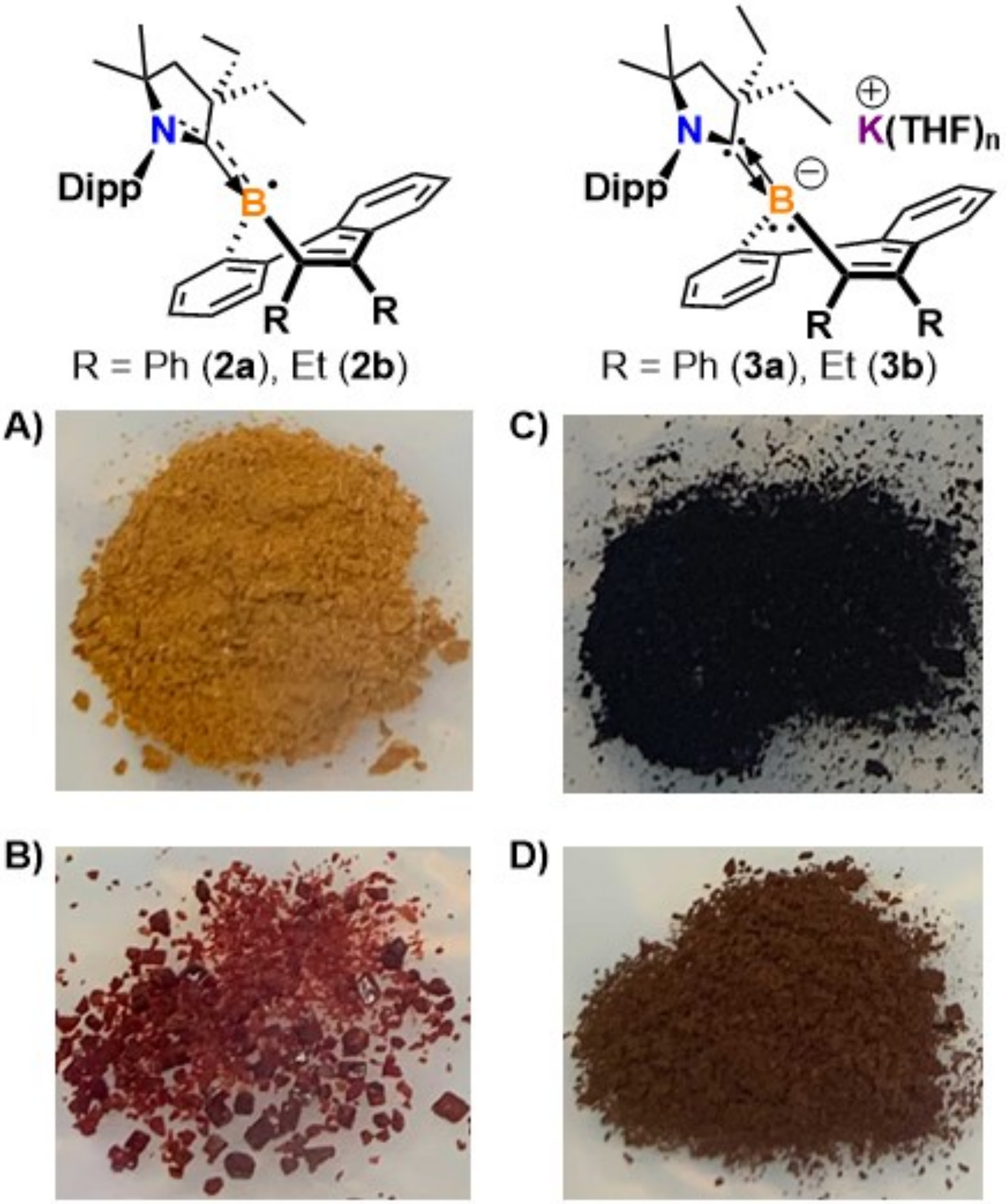

Figure S1. A) crystalline **2a**. B) crystalline **2b**. C) crystalline **3a**. D) crystalline **3b**.

EPR Spectroscopy

Table S1. Experimental EPR spectra parameter list for **2a** and **2b**.

Parameter	**2a**	**2b**
Receiver Gain	1.00e+0.004	1.00e+0.004
Phase	0	0
Harmonic	1	1
Mod. Frequency	100.000 kHz	100.000 kHz
Mod. Amplitude	1.000 G	1.000 G
Center Field	3514.000 G	3515.000 G
Sweep Width	100.000 G	150.000 G
Resolution	2048 POINT	2048 POINT
Sweep Time	20.972 s	20.972 s
Microwave Frequency	9.849000 GHz	9.839000 GHz
Microwave Power	2.002e+000 mW	1.997e+000 mW

UV Vis Spectral Data

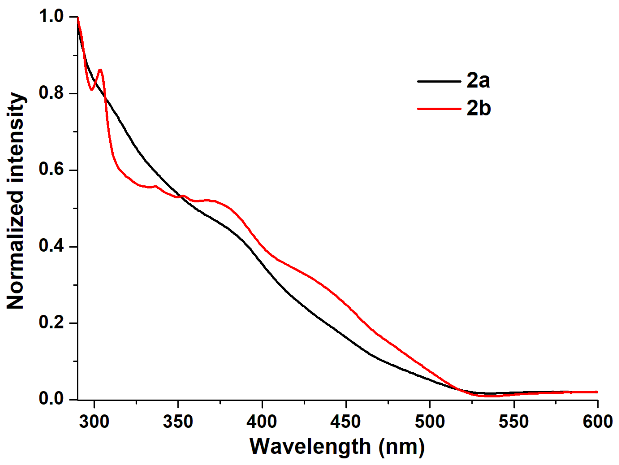

Figure S2. UV−Vis absorbance spectrum of **2a** and **2b** in toluene. The UV–vis absorption spectra of **2a** and **2b** in toluene reveal that both radicals exhibit strong absorption in the visible regions. TD-DFT calculations (ωB97XD/def2-SVP, SMD toluene) of UV-vis absorbance agree with the experimental observations (Figure S13). Bands associated with charge transfer (CAAC→borepin) were located at 294 and 328 nm for **2a**, which correspond to SOMO→LUMO+1 transitions (Figure S13). The broad bands observed in the experimental spectra may be rationalized by considering conformational flexibility of the pendant Ph and Et groups. This effect can be expected to be maximized in **2a** (R = Ph), since the LUMO+1 has significant contribution from the substituent Ph groups, and hence the SOMO→LUMO+1 transition will be impacted by conformational changes in solution.

NMR Spectral Data

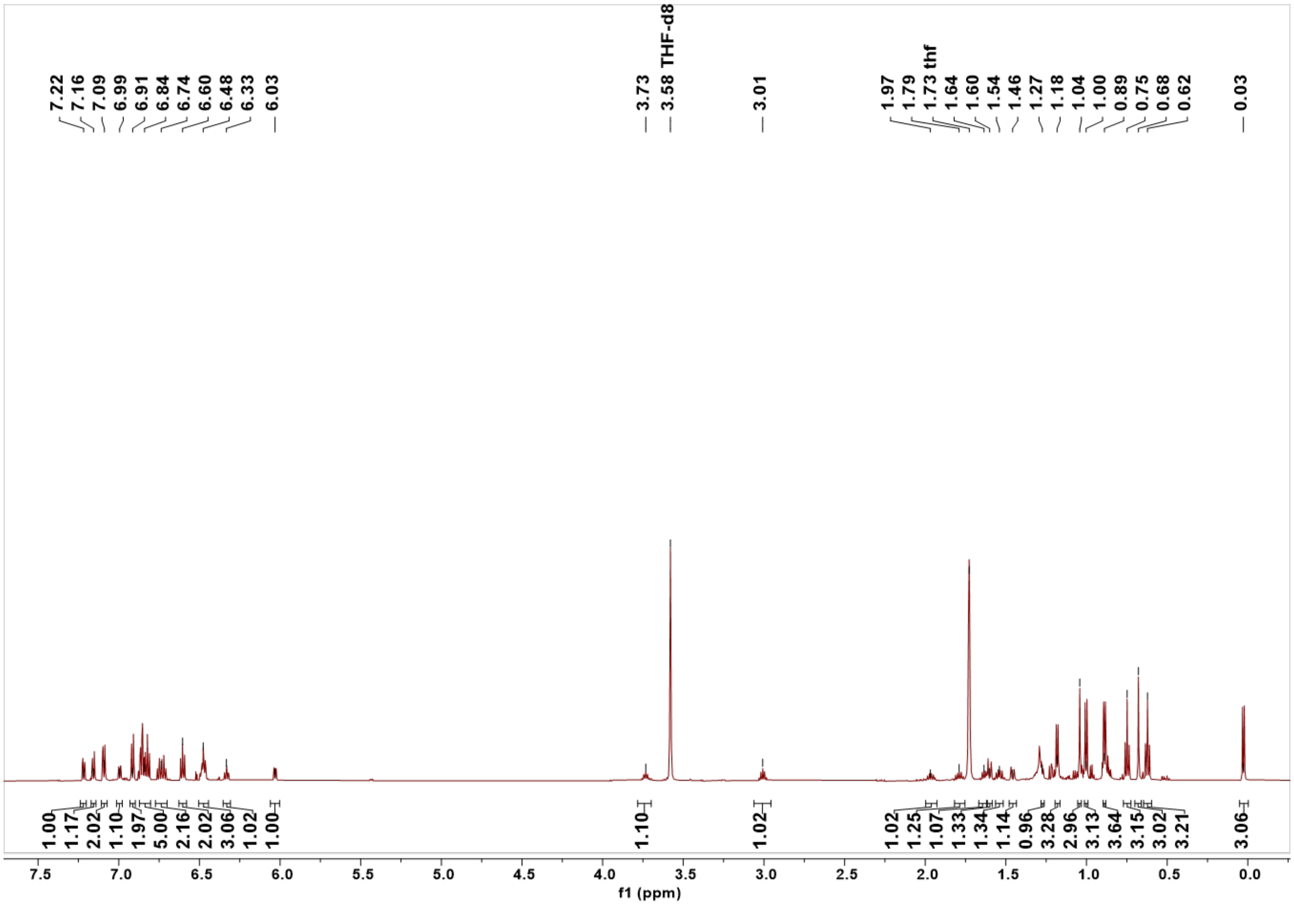

Figure S3. ^{1}H NMR spectrum (600 MHz, THF-d_8, 298 K) of **3a**.

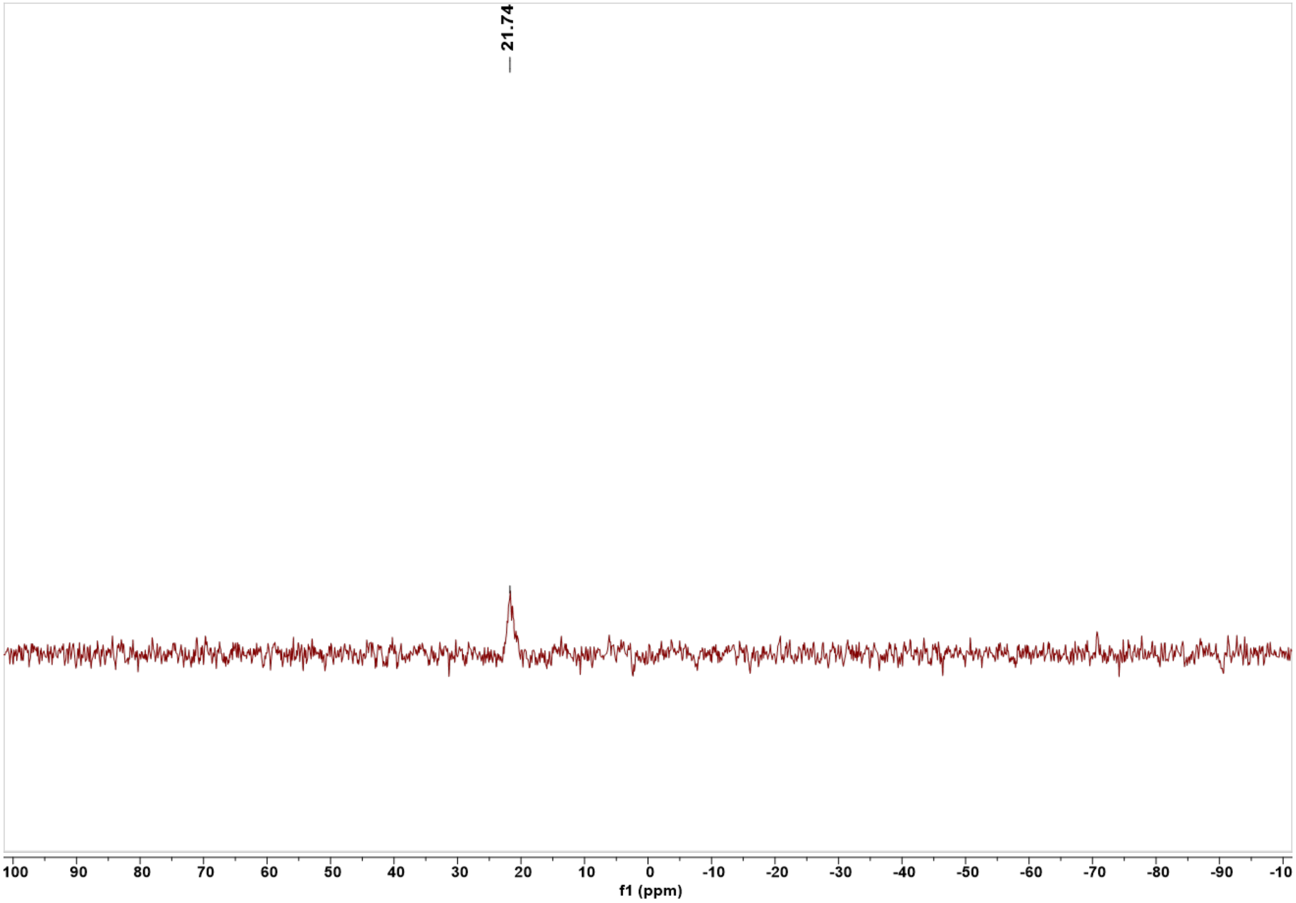

Figure S4. $^{11}B\{^{1}H\}$ NMR spectrum (192 MHz, THF-d_8, 298 K) of **3a**.

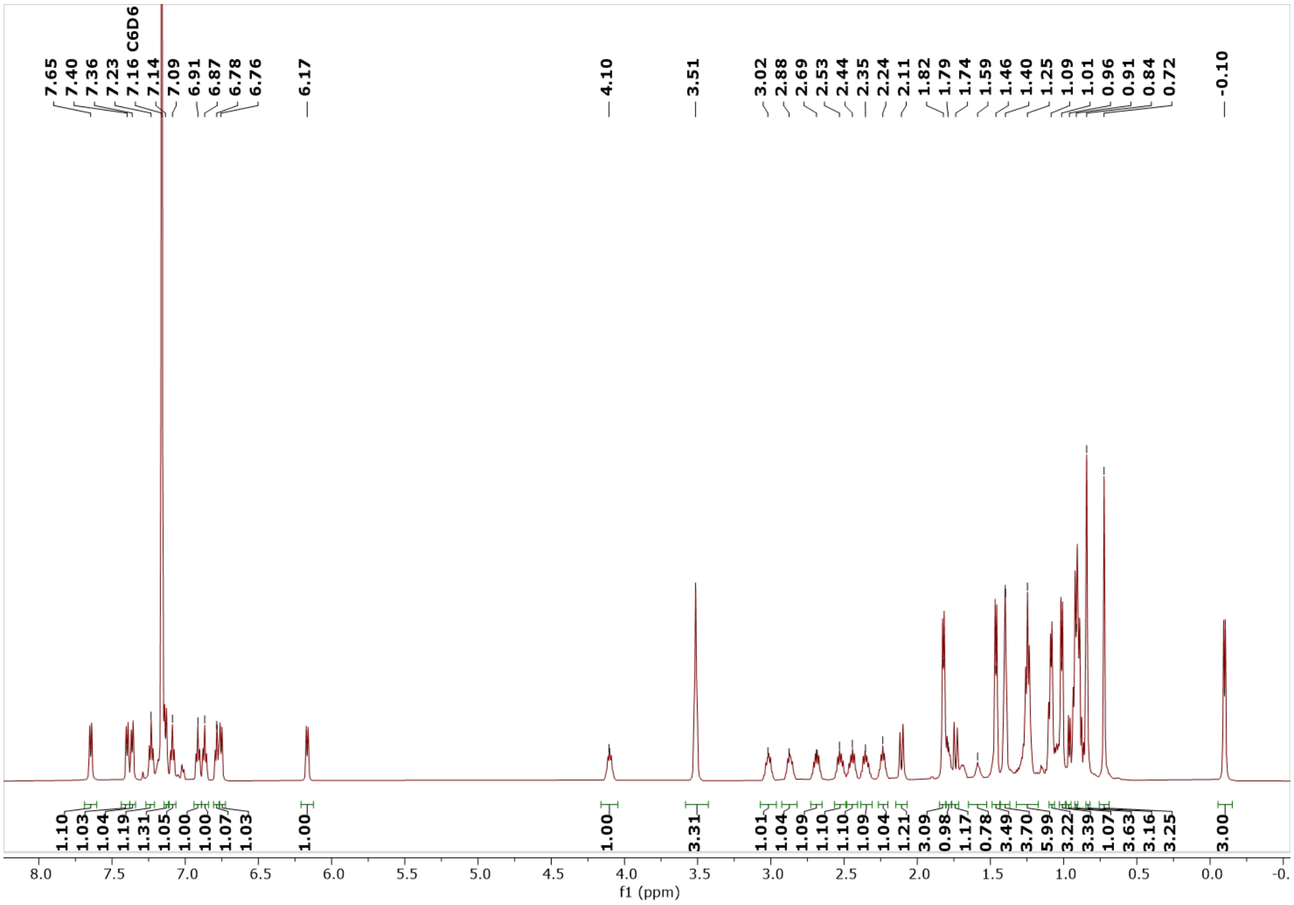

Figure S5. ^{1}H NMR spectrum (600 MHz, C_6D_6, 298 K) of **3b**.

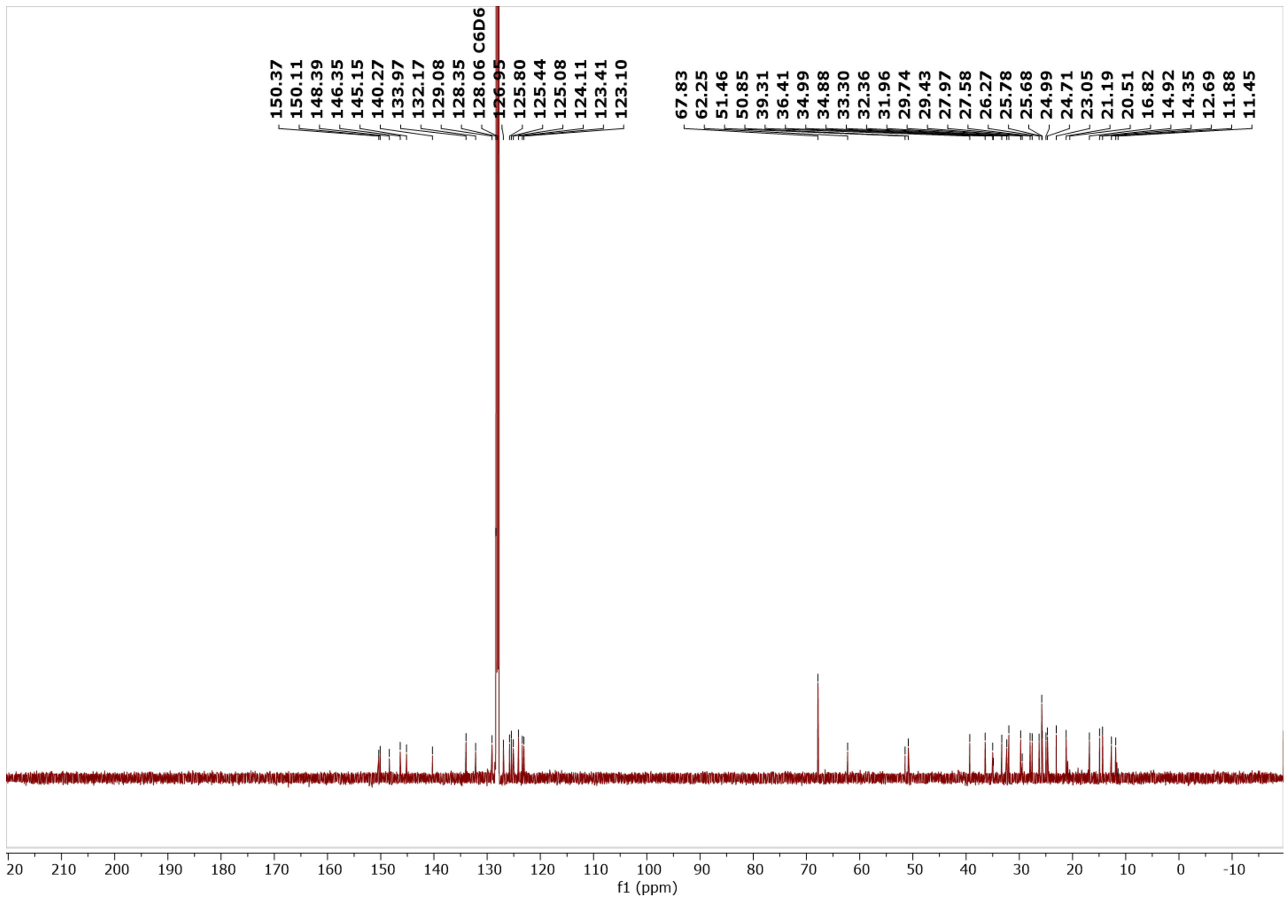

Figure S6. $^{13}C\{^{1}H\}$ NMR spectrum (201 MHz, C_6D_6, 298 K) of **3b**.

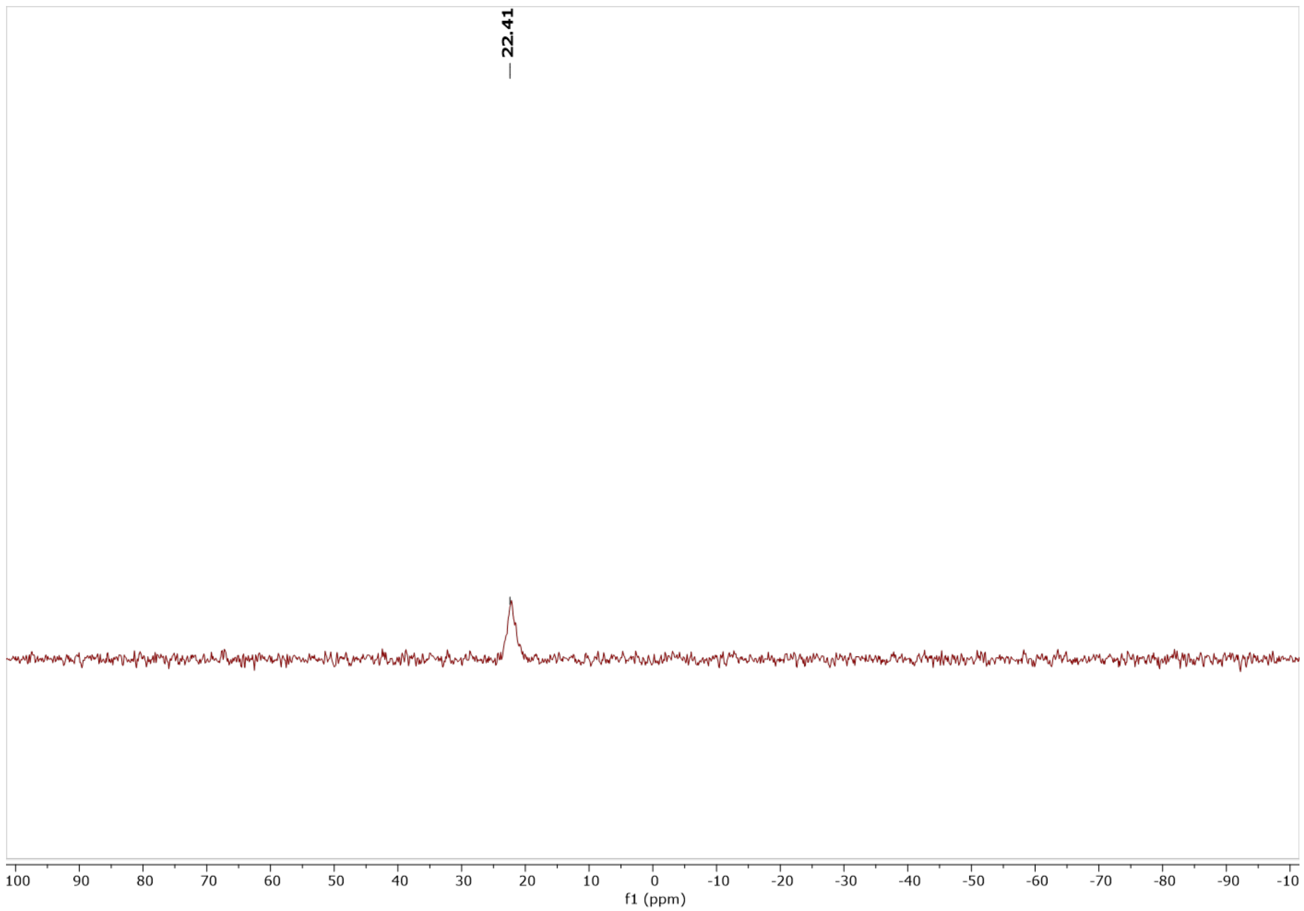

Figure S7. $^{11}B\{^{1}H\}$ NMR spectrum (192 MHz, C_6D_6, 298 K) of **3b**.

X-Ray Collection Details

Single crystals of compounds **2a**, **2b**, **3a**, **3b**, **3a^{Et_2O} and 3b^{Et_2O}** were coated with Paratone oil and mounted on a MiTeGen MicroLoop. The frames were integrated with the Bruker SAINT software package[3] using a narrow-frame algorithm. Data were corrected for absorption effects using the Multi-Scan method (SADABS or TWINABS).[3] The structures were solved and refined using the Bruker SHELXTL Software Package[4] within APEX3[3] and OLEX2.[5] Non-hydrogen atoms were refined anisotropically. Hydrogen atoms were placed in geometrically calculated positions with U_{iso} = 1.2U_{equiv} of the parent atom (U_{iso} = 1.5U_{equiv} for methyl).

In **2a**, a solvent site containing a disordered mixture of hexanes and toluene could not be adequately modeled with or without restraints. Thus, the structure factors were modified using the PLATON SQUEEZE[6] technique, in order to produce a “solvate-free” structure factor set. PLATON reported a total electron density of 224 e^- and total solvent accessible volume of 938 Å^3. In **2b**, the co-crystallized hexane solvent was disordered over three positions. The sum of the three positions was set to one and the relative occupancies were freely refined with constraints on the anisotropic displacement parameters of the disordered atoms. In compound **3a** and **3b**, the relative occupancy of the disordered atoms in the THF fragments was freely refined. Constraints and restraints were used on the anisotropic displacement parameters and bond lengths of the disordered atoms. In compound **3a^{Et_2O}**, a three domain twin was identified using CELL_NOW.[7] Starting with 1281 reflections, 772 reflections were fit to the first domain, 689 to the second domain (441 exclusively), and 667 to the third domain (81 exclusively) with 14 unindexed reflection remaining. The second domain was oriented at a 179.4° rotation about the real axis 1.000 0.001 -0.001. The twin law was 1.000 0.001 -0.001 / 0.894 -1.004 0.007 / 0.920 -0.018 -0.996. The third domain was oriented at a 178.8° rotation about the real axis 1.000 0.004 -0.003 and its twin law was 1.002 0.002 -0.008 / 0.893 -0.989 -0.018 / 0.877 0.038 -1.013. The structure was refined on HKLF5 data with the BASF for the twin domains refining to 0.36003 and 0.15721. In compound **3b^{Et_2O}**, part of one diethyl ether molecule was disordered over two positions. The relative occupancy was freely refined, and constraints were used on the anisotropic displacement parameters of the disordered atoms. Solvent located in the crystal lattice (hexanes and/or diethyl ether) was severely disordered and could not be adequately modeled with or without restraints. Thus, the structure factors were modified using the PLATON SQUEEZE[6] technique, in order to produce a “solvate-free” structure factor set. PLATON reported a total electron density of 199 e^- and total solvent accessible volume of 871 Å^3.

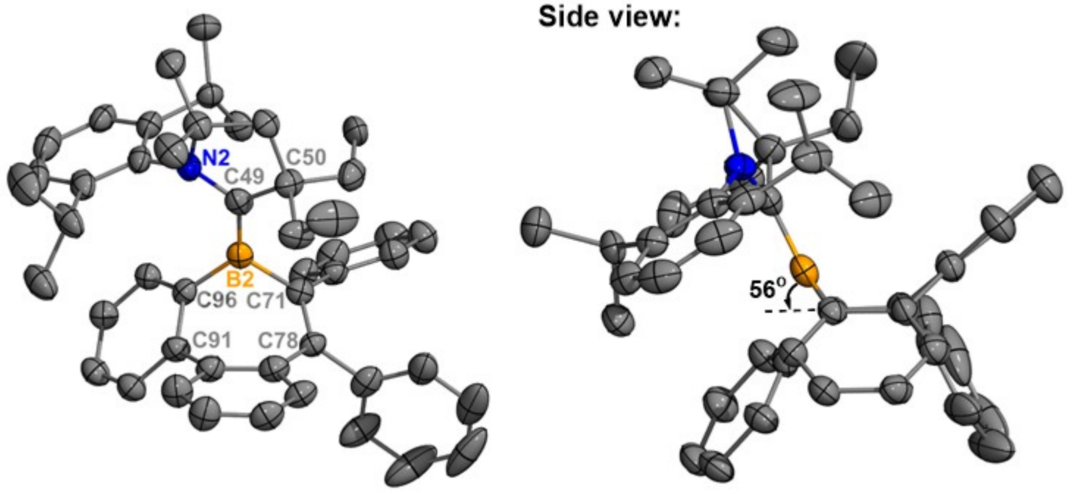

Figure S8. Molecular structure of **2a** (thermal ellipsoids at 50% probability; H atoms were omitted for clarity, only one of two crystallographically independent molecules shown (second structure in Figure 1). Selected bond lengths [Å] and angles [°] - 2a: B2–C49 1.5263(57), B2–C71 1.6061(55), B2–C96 1.5764(55), C49–B2–C96 127.8(4), C49–B2–C71 123.7(3), C71–B2–C96 107.6(3).

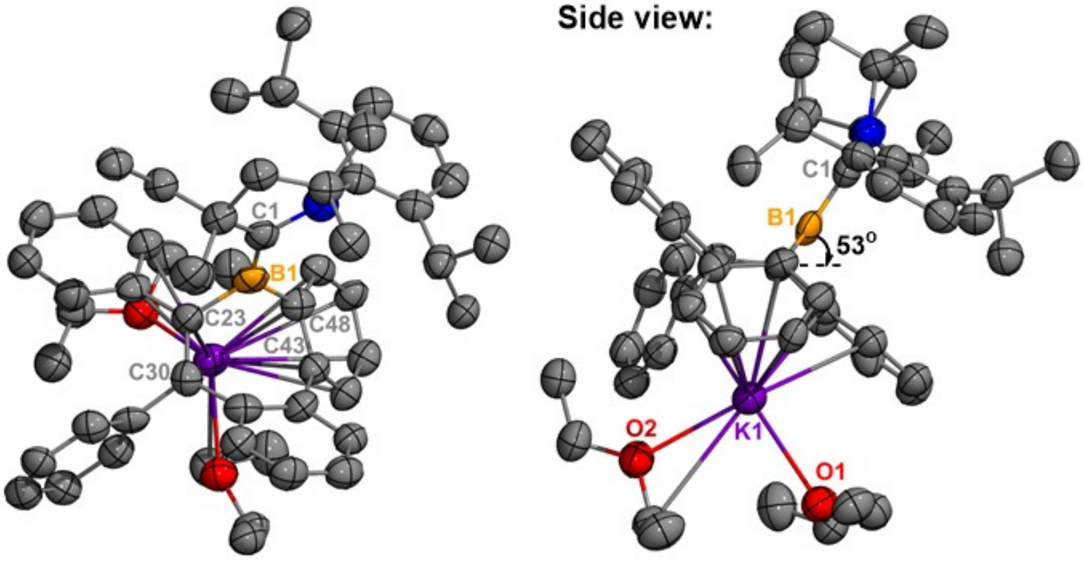

Figure S9. Molecular structure of **$3a^{Et_2O}$** (thermal ellipsoids at 50% probability; H atoms were omitted for clarity). The boron center lies 0.7591 Å above the plane containing C30−C23−C48−C43. Selected bond lengths [Å] and angles [°] – **$3a^{Et_2O}$**: B1–C1 1.443(12), B1–C23 1.620(12), B1–C48 1.609(12), C1-B1-C23 124.1(7), C1-B1-C48 128.5(8), C23-B1-C48 107.2(6). Note: The reaction was run by the addition of ^{Et}CAAC and KC_8 to an Et_2O solution of **1a**. The crystals were grown from a Et_2O/hexanes mixture at -37°C. Only structural data was able to be obtained in this solvent system.

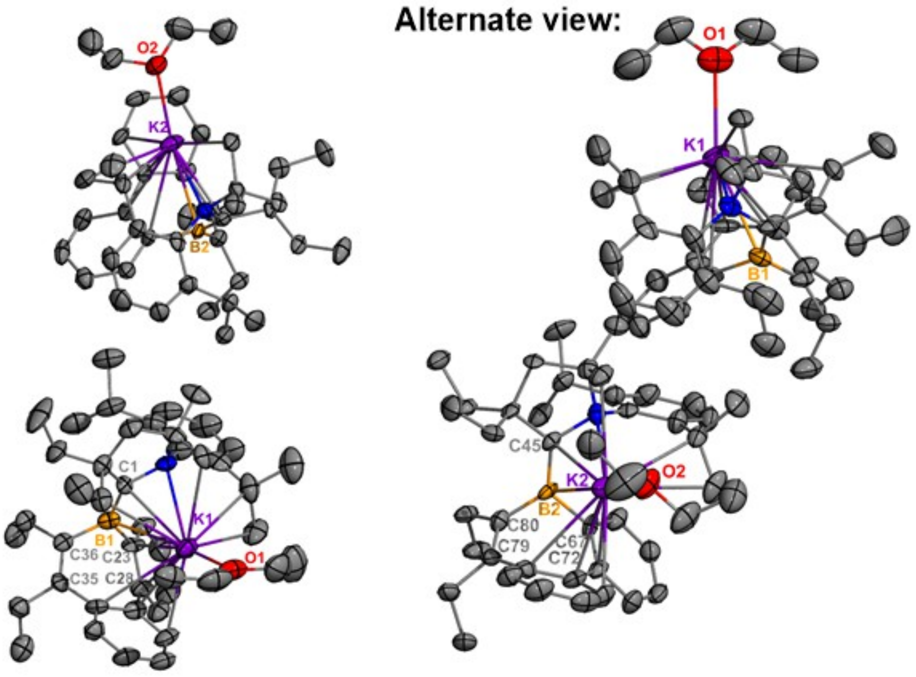

Figure S10. Molecular structure of $3b^{Et_2O}$ (thermal ellipsoids at 50% probability; H atoms were omitted for clarity, two crystallographically independent molecules shown). The boron centers lie 0.7244 Å and 0.7488 Å above the plane containing C35−C36−C23−C28 and C79−C80−C67−C72, respectively. Selected bond lengths [Å] and angles [°] - $3b^{Et_2O}$: B1–C1 1.471(11), B1–C23 1.585(12), B1–C36 1.617(13), C1-B1-C23 127.3(8), C1-B1-C36 125.3(8), C23-B1-C36 107.4(7), B2–C45 1.492(11), B2–C67 1.606(11), B2–C80 1.594(12), C45–B2–C67 128.9(7), C45–B2–C80 124.2(7), C67–B2–C80 106.8(6). Note: The reaction was run by the addition of ^{Et}CAAC and KC_8 to an Et_2O solution of **1b**. The crystals were grown from a Et_2O/hexanes mixture at -37°C. Compound **3b** proved to be less stable in Et_2O relative to THF; therefore, suitable NMR was not able to be obtained.

Table S2. Crystal data table for compounds **2a**, **2b**, **3a, 3a, 3b, $3a^{Et_2O}$ and $3b^{Et_2O}$**

	**2a**	**2b**	**3a**	**3b**	**$3a^{Et_2O}$**	**$3b^{Et_2O}$**
CCDC number	2017226	2017227	2061342	2112405	2061341	2061343
Chemical formula	$C_{48}H_{53}BN$	$C_{43}H_{60}BN$	$C_{76}H_{109}BKNO_7$	$C_{48}H_{69}BKNO_2$	$C_{56}H_{72}BKNO_2$	$C_{44}H_{63}BKNO$
F_w	654.72	601.66	1198.55	741.95	841.05	671.86
Crystal system	monoclinic	triclinic	monoclinic	orthorhombic	triclinic	orthorhombic
Space group	P 2_1/c	P -1	P 2_1/n	P$2_12_12_1$	P -1	P ca2_1
Temperature (K)	100(2)	100(2)	100(2)	100(2)	100(2)	100(2)
a (Å)	36.702(2)	9.3590(11)	11.1653(11)	10.5016(2)	10.1913(14)	21.410(3)
b (Å)	11.0451(8)	10.2125(12)	23.217(2)	20.1270(4)	13.5452(19)	10.1549(12)
c (Å)	20.2219(14)	19.287(2)	26.583(3)	20.4743(4)	20.993(3)	39.099(5)
α (°)	90	97.762(3)	90	90	63.242(9)	90
β (°)	94.653(4)	95.537(3)	91.691(3)	90	77.081(8)	90
γ (°)	90	96.018(4)	90	90	70.259(8)	90
V (Å^3)	8170.5(10)	1805.0(4)	6888.0(11)	4327.56(15)	2426.7(6)	8500.8(18)
Z	8	2	4	4	2	8
μ (mm^{-1})	0.447	0.062	0.130	1.139	1.261	0.156
Crystal size (mm)	0.028 x 0.033 x 0.143	0.144 x 0.224 x 0.396	0.272 x 0.276 x 0.687	0.06 × 0.065 × 0.072	0.057 x 0.078 x 0.165	0.284 x 0.394 x 0.452
T_{min}, T_{max}	0.9390, 0.9880	0.9760, 0.9910	0.9160, 0.9650	0.909, 0.923	0.8190, 0.9320	0.9330, 0.9570
No. of measured, independent and observed [$I > 2\sigma(I)$] reflections	46262, 14587, 7316	42860, 10132, 7117	80845, 14156, 9982	47611, 7918, 7234	8840, 4263, 3308	77057, 14054, 9958
R_{int}	0.1424	0.0460	0.0581	0.0620	0.1291	0.0810
R[$F^2 > 2\sigma(F^2)$], wR(F^2), S	0.0753, 0.2141, 0.947	0.0534, 0.1413, 1.020	0.0841, 0.1978, 1.127	0.0378, 0.0987, 1.034	0.1237, 0.3834, 1.088	0.0846, 0.2089, 1.065
Data/Restraints/Parameters	14587 / 0 / 917	10132 / 1 / 428	14156 / 84 / 839	7918/166/562	8840 / 0 / 564	14054 / 2 / 893
Δ>max, Δ>min (e Å-3)	0.220, -0.276	0.506, -0.557	0.968, -0.545	0.22/-0.14	0.844, -0.438	0.340, -0.286
Absolute structure parameter	-	-	-	0.007(4)	-	0.08(4)

Theoretical Calculations

Geometry optimizations and subsequent analytical harmonic frequency calculations for compounds **2a, 2b, 3a** and **3b** were carried out at the B3LYP-D3(BJ)[8]/def2-TZVP[9] level. For the anionic complex, the inclusion of diffuse basis functions is usually recommended. However, because of the large size of **3a**, we could not use a larger basis set. However, we optimized **3b** at the B3LYP-D3(BJ)/def2-TZVPD level, but the corresponding geometrical parameters remain more or less the same. Thereafter, we continue with the B3LYP-D3(BJ)/def2-TZVP level. TD-DFT calculations employed ωB97XD/def2-SVP[9] (solvation with SMD, toluene),[10] with 20 singlet states. Wiberg bond indices were computed using NBO 6.0. EPR parameters were computed at the B3LYP/EPR-II//B3LYP-D3(BJ)/def2-TZVP level[10] using ORCA 4.2.0 to obtain the hyperfine coupling values.[11] The EPR spectra were simulated using EasySpin.[12] Atomic partial charges were computed using the Hirshfeld-CM5 scheme due to its basis-set independence and accurate description for polarization in ionic compounds.[13]

The energy decomposition analysis (EDA) in combination with the natural orbital for chemical valence (NOCV) method was performed at the B3LYP-D3(BJ)/TZ2P//B3LYP-D3(BJ)/def2-TZVP level using the ADF (2018.105) program package.[14]

In the EDA method, the interaction energy (ΔE_{int}) between two prepared fragments is divided into three energy terms, viz., the electrostatic interaction energy (ΔE_{elstat}), which represents the quasiclassical electrostatic interaction between the unperturbed charge distributions of the prepared atoms, the Pauli repulsion (ΔE_{Pauli}), which is the energy change associated with the transformation from the superposition of the unperturbed electron densities of the isolated fragments to the wavefunction that properly obeys the Pauli principle through explicit antisymmetrization and renormalization of the product wavefunction, and the orbital interaction energy (ΔE_{orb}), which is originated from the mixing of orbitals, charge transfer and polarization between the isolated fragments. Lastly, since we have used dispersion corrected functional, it adds the dispersion contribution to the interaction energy. Therefore, the interaction energy (ΔE_{int}) between two fragments can be defined as:

$$\Delta E_{int} = \Delta E_{elstat} + \Delta E_{Pauli} + \Delta E_{orb} + \Delta E_{disp} \quad (1)$$

The EDA-NOCV calculation combines charge and energy decomposition schemes to divide the deformation density, $\Delta\rho(\mathbf{r})$, associated with the bond formation into different components (σ, π, δ) of a chemical bond. From the mathematical point of view, each NOCV, ψ_i is defined as an eigenvector of the deformation density matrix in the basis of fragment orbitals.

$$\Delta P \Psi_i = v_i \Psi_I \quad (2)$$

In EDA-NOCV, ΔE_{orb} is given by the following equation

$$\Delta E_{orb} = \sum_{k} \Delta E_{k}^{orb} = \sum_{k=1}^{\frac{N}{2}} v_k \left[-F_{-k}^{TS} + F_{k}^{TS}\right] \quad (3)$$

where, $-F_{-k}^{TS}$ and F_{k}^{TS} are diagonal Kohn-Sham matrix elements corresponding to NOCVs with the eigenvalues $-v_k$ and v_k, respectively. The ΔE_{k}^{orb} terms are assigned to a particular type of bond by visual inspection of the shape of the deformation density, $\Delta\rho_k$. The EDA-NOCV scheme thus provides both qualitative ($\Delta\rho_{orb}$) and quantitative (ΔE_{orb}) information about the strength of orbital interactions in chemical bonds. More details about EDA-NOCV and its application can be found in recent reviews.[15]

Cartesian coordinates optimized at the B3LYP-D3(BJ)/def2-TZVP level of theory

2a

E = -1941.595160 a.u

B	0.08937900	-0.12013200	-0.25550100
N	-2.16575300	-0.25440000	0.95260700
C	-0.80753400	-0.43374100	0.91995700
C	-0.33164100	-0.84253100	2.32405400
C	-1.65821100	-1.12237500	3.05829900
H	-1.90622400	-2.18031500	2.95233900
H	-1.62418500	-0.90726600	4.12272100
C	-2.72584700	-0.29080300	2.34521600
C	-2.79775000	1.11796200	2.95249400
H	-1.86260000	1.65723900	2.81781000
H	-2.99398500	1.03533000	4.02272000
H	-3.59803600	1.70639500	2.51628200
C	-4.10129900	-0.94158600	2.39877100
H	-4.83333900	-0.38717900	1.81252200
H	-4.44312300	-0.96243100	3.43448700
H	-4.07456000	-1.96477000	2.03075400
C	0.51821100	0.31079400	2.94238600
H	1.55075800	0.14802300	2.63237700
H	0.23495300	1.25198200	2.47243500
C	0.47121800	0.50297200	4.45703500
H	0.69244900	-0.41045500	5.00775600
H	-0.50213800	0.86291700	4.79203200
H	1.20779900	1.25386600	4.75016000
C	0.49872700	-2.13781000	2.22886600
H	-0.09131600	-2.88503600	1.69304800
H	1.36669600	-1.94358300	1.60904000
C	0.96829500	-2.73218400	3.55419800
H	1.45613900	-3.69243900	3.37703000
H	0.14120000	-2.90580000	4.24580600
H	1.69228700	-2.08760300	4.05296800
C	-2.99655000	-0.38217500	-0.21104300
C	-3.64238700	0.73234600	-0.77137000
C	-4.43268500	0.54307000	-1.90310700
H	-4.92451600	1.39561700	-2.35101000
C	-4.58009100	-0.70511700	-2.47962800
H	-5.18854900	-0.82743800	-3.36661400
C	-3.94841800	-1.79849000	-1.91157000
H	-4.07259000	-2.77602200	-2.35821600
C	-3.16127800	-1.66664200	-0.77100500
C	-2.52622500	-2.91662800	-0.18729100
H	-2.08781700	-2.65284800	0.77050000
C	-1.39312800	-3.43882800	-1.07570700
H	-1.76568800	-3.71621200	-2.06416100
H	-0.93024000	-4.32203000	-0.63107200
H	-0.61485900	-2.69110700	-1.20558400
C	-3.55811600	-4.02172600	0.07210600
H	-4.39501100	-3.66125300	0.67153600
H	-3.08928500	-4.85226000	0.60356900
H	-3.96586000	-4.41771500	-0.85924200
C	-3.51945500	2.13172100	-0.20395500
H	-2.73261900	2.11425400	0.54747900
C	-3.11522800	3.16230500	-1.26275200
H	-2.21752900	2.85863200	-1.79105700
H	-2.92721800	4.12676800	-0.78615100
H	-3.90703900	3.31193600	-1.99874200
C	-4.83665800	2.56612000	0.45656800
H	-5.61770900	2.68222700	-0.29752400
H	-4.71503300	3.52634400	0.96259800
H	-5.19531300	1.83880200	1.18450800
C	-0.28280500	0.71065600	-1.53819500
C	-0.95126800	0.23869600	-2.67035000
H	-1.39718400	-0.74562700	-2.65126500
C	-1.06219100	1.00396200	-3.82281600
H	-1.58947500	0.61011300	-4.68255600
C	-0.49596100	2.27218400	-3.86860800
H	-0.56437000	2.87170900	-4.76727500
C	0.15382000	2.77180100	-2.74893800
H	0.59004500	3.76230100	-2.77687000
C	0.26694300	2.00976900	-1.58279900
C	0.91439000	2.61841500	-0.39285500
C	0.47980800	3.89194500	-0.00160600
H	-0.33584200	4.34711900	-0.54718200
C	1.05257900	4.56728200	1.06270700
H	0.68128200	5.54309300	1.34858700
C	2.11256100	3.98548800	1.74941400
H	2.58360400	4.50450800	2.57431400
C	2.56629400	2.73494500	1.36956600
H	3.40128500	2.29294200	1.89478100
C	1.96713700	2.01177200	0.33048200
C	2.48898300	0.66676800	-0.01108600
C	3.97384300	0.55304700	-0.12155500
C	4.76851700	0.20343100	0.96964400
H	4.29841700	-0.00627900	1.92258200
C	6.14854500	0.10275400	0.84262400
H	6.74936600	-0.17559600	1.69932100
C	6.75474200	0.35022800	-0.38288200
H	7.82906500	0.26759000	-0.48545100
C	5.97221600	0.70124600	-1.47705200
H	6.43604900	0.89085800	-2.43676900
C	4.59381100	0.80622700	-1.34477400
H	3.98295100	1.07481800	-2.19713400
C	1.66111600	-0.36169600	-0.28098600
C	2.18140300	-1.64678500	-0.82394200
C	1.78222600	-2.05544600	-2.10145000
H	1.12035800	-1.42208500	-2.67495600
C	2.24350900	-3.24348800	-2.65202500
H	1.92421300	-3.53067600	-3.64605800
C	3.11175900	-4.05780700	-1.93566300
H	3.47052500	-4.98553700	-2.36241700
C	3.52122100	-3.66354100	-0.66760000
H	4.20447100	-4.28316100	-0.10043900
C	3.06336900	-2.47242800	-0.12125300
H	3.39960800	-2.17768000	0.86180000

2b

E = -1636.606041 a.u

B	-0.57694800	0.13339100	-0.74133300
N	1.52349100	0.69542600	0.62495800
C	0.33280200	1.05042900	0.04487200
C	-0.01765300	2.48327200	0.48262200
C	1.30128700	2.96318700	1.11934200
H	1.91437700	3.43518200	0.34844700
H	1.16569900	3.69299800	1.91305300
C	2.01924800	1.70714500	1.61633200
C	3.53461800	1.85425800	1.58349400
H	4.03570100	0.92539200	1.85468200
H	3.83190600	2.62098700	2.30031000
H	3.88566900	2.15664000	0.59964500
C	1.57287100	1.36109500	3.04438700
H	0.51301300	1.12085400	3.08512100
H	1.75416300	2.21995300	3.69265200
H	2.13007000	0.52214300	3.44778500
C	-0.35526600	3.35536300	-0.74535300
H	0.45149700	3.24241400	-1.47455100
H	-1.25335700	2.95887100	-1.21229800
C	-0.56652000	4.84299300	-0.46958800
H	0.27627800	5.28875500	0.06237500
H	-1.46652400	5.02075000	0.11852900
H	-0.68292900	5.38307900	-1.41110200
C	-1.23105000	2.43486900	1.46403100
H	-2.13367000	2.48042000	0.85477700
H	-1.26781400	1.45307400	1.93561900
C	-1.30896100	3.49362200	2.56262100
H	-0.52333000	3.36732700	3.30825900
H	-2.26352700	3.40321600	3.08508100
H	-1.23753500	4.51039100	2.17864800
C	2.37519300	-0.32979600	0.09392900
C	3.03225900	-0.08569800	-1.13062700
C	3.86862300	-1.07200500	-1.64565800
H	4.37235800	-0.89984800	-2.58764500

C	4.06841400	-2.26749000	-0.97618500
H	4.72248600	-3.02276200	-1.39286500
C	3.42469000	-2.49009700	0.22693700
H	3.57240200	-3.42953800	0.74193300
C	2.56934300	-1.53964000	0.78070200
C	1.88347300	-1.86659300	2.09137500
H	1.15513200	-1.08240700	2.28647000
C	2.89884500	-1.90749900	3.24310600
H	2.38850300	-2.02085200	4.20198100
H	3.51278800	-1.00786800	3.28587900
H	3.57554900	-2.75589000	3.12302900
C	1.11851900	-3.19288000	2.03263900
H	1.79706100	-4.04129200	1.92846000
H	0.42358500	-3.21344800	1.19949900
H	0.55502300	-3.33584700	2.95721700
C	2.86066900	1.19524300	-1.92815800
H	2.27159700	1.88678100	-1.33264500
C	2.08598400	0.93616100	-3.22577600
H	2.64363500	0.27045600	-3.88761200
H	1.90978600	1.87160900	-3.76093000
H	1.12125200	0.47595000	-3.02289000
C	4.20042500	1.87477900	-2.23779700
H	4.80683000	1.27368500	-2.91714700
H	4.78740000	2.04297800	-1.33452800
H	4.02882900	2.84041700	-2.71781100
C	-1.89287100	0.60676500	-1.47773400
C	-3.09234600	0.36806900	-0.91342700
C	-3.17331500	-0.23911200	0.43975100
C	-4.03126200	0.32410700	1.39299200
H	-4.60650400	1.19993100	1.12361200
C	-4.14258400	-0.18228400	2.67636500
H	-4.79785600	0.29689700	3.39267000
C	-3.40418700	-1.30397300	3.03658000
H	-3.47494300	-1.71175900	4.03682400
C	-2.57382900	-1.89779900	2.10104300
H	-1.99683500	-2.77104200	2.37526400
C	-2.43803500	-1.38987100	0.80234400
C	-1.54064200	-2.11459500	-0.13132300
C	-0.51414800	-1.43845600	-0.82622000
C	0.33920800	-2.19585700	-1.63211800
H	1.14156800	-1.70706000	-2.16524100
C	0.19032200	-3.56962600	-1.76533100
H	0.87225400	-4.12625800	-2.39587800
C	-0.83141500	-4.22456300	-1.08924800
H	-0.96288400	-5.29385400	-1.19487500
C	-1.68409800	-3.49696400	-0.27101300
H	-2.48069900	-4.00146100	0.26128600
C	-1.75011000	1.12055300	-2.89902800
H	-2.50906700	1.87415400	-3.12346500
H	-0.78963500	1.62526000	-3.00723400
C	-1.83158500	-0.00452300	-3.93851200
H	-1.08569000	-0.77485600	-3.74112700
H	-1.66618600	0.38307600	-4.94648000
H	-2.80911800	-0.48679600	-3.91413600
C	-4.42172200	0.63381100	-1.58387800
H	-5.04151900	1.27700400	-0.95189500
H	-4.27339600	1.17894500	-2.51441900
C	-5.19374500	-0.65989000	-1.87067100
H	-4.61278200	-1.32175900	-2.51502500
H	-6.14408600	-0.44768600	-2.36476300
H	-5.40490100	-1.20130300	-0.94751200

3a
E = -1941.643654 a.u

B	0.09584300	-0.08270400	-0.24071300
N	-2.20698700	-0.31048400	0.88366400
C	-0.79405900	-0.59716700	0.78074600
C	-0.38731000	-1.34473200	2.07521600
C	-1.73477200	-1.54693900	2.80042700
H	-2.14384000	-2.52262900	2.52644800
H	-1.66255900	-1.52854900	3.88690600
C	-2.68670700	-0.46173300	2.26644400
C	-2.51591600	0.83172800	3.08846500
H	-1.52686700	1.25640900	2.92587000
H	-2.63384600	0.62605300	4.15604300
H	-3.25435700	1.58077200	2.81182300
C	-4.13801500	-0.92690400	2.34329000
H	-4.82061000	-0.20467600	1.89655100
H	-4.42348100	-1.06283000	3.38899000
H	-4.27203300	-1.87565800	1.82498000
C	0.63067500	-0.48281600	2.88912800
H	1.62988600	-0.73082200	2.52422300
H	0.48786000	0.55883700	2.60887500
C	0.62121900	-0.57485900	4.41701200
H	0.69521800	-1.59785200	4.78689300
H	-0.28475600	-0.13797800	4.84049100
H	1.46704700	-0.01368500	4.82482500
C	0.23987800	-2.71657900	1.74316700
H	-0.48685500	-3.29303800	1.16907400
H	1.08123600	-2.55798300	1.07515600
C	0.71067200	-3.56945000	2.92233200
H	1.04595800	-4.54829500	2.56879800
H	-0.08644000	-3.73638200	3.65189300
H	1.55052000	-3.11007200	3.44610700
C	-3.05820200	-0.30172500	-0.24166200
C	-3.71152800	0.88834500	-0.63762300
C	-4.55222400	0.87559100	-1.74956200
H	-5.04297600	1.79275100	-2.05131200
C	-4.73719300	-0.26958900	-2.50277700
H	-5.37966500	-0.25612800	-3.37519500
C	-4.08788000	-1.43665700	-2.12471600
H	-4.23727300	-2.34013200	-2.70447000
C	-3.26626900	-1.47816100	-1.00445400
C	-2.64033500	-2.80081500	-0.61643900
H	-2.12302100	-2.63600800	0.32071200
C	-1.59278100	-3.25750300	-1.63442600
H	-2.04185200	-3.42746600	-2.61703100
H	-1.12088100	-4.18951600	-1.31303200
H	-0.81070600	-2.50756100	-1.72632600
C	-3.69267000	-3.89210300	-0.38504900
H	-4.44515200	-3.57254500	0.33819700
H	-3.21659100	-4.79960900	-0.00402100
H	-4.21283600	-4.15443100	-1.30930600
C	-3.48081400	2.20314600	0.07999900
H	-2.65135900	2.03844800	0.76492400
C	-3.07112500	3.32500200	-0.88032600
H	-2.22902100	3.02862600	-1.49760100
H	-2.78550300	4.21480800	-0.31296300
H	-3.89382900	3.60833200	-1.54162700
C	-4.71420900	2.64360900	0.88014200
H	-5.55067000	2.85107100	0.20724400
H	-4.50314700	3.55704800	1.44342300
H	-5.04200400	1.88028800	1.58466900
C	-0.23421500	0.94076400	-1.40645300
C	-0.93020100	0.63735200	-2.58424700
H	-1.40469300	-0.33029500	-2.67411900
C	-1.04076400	1.53801200	-3.63505500
H	-1.59502700	1.26301300	-4.52503900
C	-0.44042200	2.78967500	-3.54284600
H	-0.50575100	3.49552500	-4.36222200
C	0.23338500	3.13231600	-2.37805000
H	0.68969500	4.11137400	-2.28939300
C	0.33292900	2.23250700	-1.31228900
C	0.97389400	2.68891900	-0.05429000
C	0.54746800	3.90989300	0.48408000
H	-0.25433700	4.43698300	-0.01607800
C	1.09848900	4.43698800	1.64088800
H	0.72774800	5.37258200	2.04195900
C	2.12469000	3.75049000	2.27930300
H	2.57183200	4.14471900	3.18391600
C	2.57161100	2.54977300	1.75280200
H	3.37602800	2.02546700	2.24918700
C	2.00043500	1.97902100	0.60887300
C	2.52144300	0.68856900	0.09390000

C	4.00414900	0.61633300	-0.08343800
C	4.86790500	0.26893700	0.95690900
H	4.45282800	0.02290500	1.92620300
C	6.24141400	0.20147000	0.75783000
H	6.89075800	-0.07996900	1.57834400
C	6.78055900	0.48083500	-0.49251100
H	7.85032700	0.42279400	-0.65168500
C	5.93258200	0.82752600	-1.53854800
H	6.34029900	1.03986800	-2.51961300
C	4.56090500	0.89793000	-1.33262600
H	3.89840300	1.16043000	-2.14740800
C	1.68702600	-0.30413300	-0.27977200
C	2.23176700	-1.53061300	-0.91353600
C	1.79672500	-1.88940800	-2.19518600
H	1.08570800	-1.25005000	-2.69984600
C	2.26862200	-3.03408700	-2.82111000
H	1.91772100	-3.28206500	-3.81584100
C	3.17859000	-3.86574000	-2.17499800
H	3.53923400	-4.76549400	-2.65843400
C	3.61476400	-3.52882000	-0.89956900
H	4.31930900	-4.16754400	-0.37991400
C	3.15208600	-2.37372900	-0.28177500
H	3.49638000	-2.12575600	0.71194100

3b
E = -1636.648161 a.u

B	-0.61134900	0.26674600	-0.64248600
N	1.53871100	0.42984800	0.76741000
C	0.35033700	0.99607800	0.16226200
C	0.20570000	2.43981400	0.70560600
C	1.49156600	2.62668200	1.53575400
H	2.26510700	3.08704400	0.91853400
H	1.35552200	3.27050100	2.40660400
C	1.98926100	1.22037900	1.92252400
C	3.50802400	1.22184000	2.08578400
H	3.89533300	0.22133300	2.27643400
H	3.78325800	1.85940500	2.92942900
H	3.99905400	1.60415600	1.19183600
C	1.34822100	0.75261500	3.24719700
H	0.30397700	0.48957800	3.09508600
H	1.39996900	1.54236700	4.00208400
H	1.86026800	-0.12149500	3.64578300
C	0.10745300	3.49631200	-0.43334400
H	0.66671600	3.12523000	-1.29131900
H	-0.93362200	3.54089600	-0.75968400
C	0.59791200	4.91894000	-0.13879500
H	1.67371900	4.94263400	0.04470900
H	0.11098300	5.36685200	0.72620500
H	0.40472900	5.56689200	-0.99903600
C	-1.07021000	2.51254600	1.58541000
H	-1.90378900	2.22253700	0.94862100
H	-1.00894100	1.73658600	2.34566800
C	-1.41360800	3.83941800	2.26582400
H	-0.58452300	4.23459500	2.85909300
H	-2.25989700	3.69770300	2.94375500
H	-1.70350300	4.60756200	1.54678700
C	2.37788500	-0.46222100	0.07099500
C	3.02986500	-0.07265300	-1.12618600
C	3.84219800	-0.98065800	-1.79453500
H	4.33679400	-0.67420300	-2.70915400
C	4.05014400	-2.26396800	-1.30591300
H	4.69205000	-2.95675900	-1.83696200
C	3.42324100	-2.64448600	-0.13307800
H	3.56387000	-3.65160800	0.24065800
C	2.57772100	-1.77629000	0.55540400
C	1.84353400	-2.29353500	1.77628800
H	1.12854400	-1.52235600	2.05366600
C	2.79296800	-2.54342200	2.95536100
H	2.23260000	-2.84109100	3.84620500
H	3.37896300	-1.65897700	3.20416000
H	3.49554100	-3.34717600	2.71863300
C	1.04778700	-3.56862900	1.47616100
H	1.70743700	-4.41050900	1.25065700
H	0.38158800	-3.42671600	0.63096300
H	0.44489100	-3.84538700	2.34556500
C	2.88820500	1.32415200	-1.69525400
H	2.26655400	1.88218100	-1.00536600
C	2.16568400	1.31170500	-3.04551800
H	2.73824000	0.76206900	-3.79739900
H	2.01873900	2.33075000	-3.41407300
H	1.18739100	0.84573900	-2.94031200
C	4.23709300	2.04520600	-1.80569400
H	4.89612900	1.56077100	-2.53009400
H	4.75728400	2.06261600	-0.84655300
H	4.08788100	3.07855000	-2.13086000
C	-1.93100300	0.89143700	-1.29612000
C	-3.14479200	0.60289300	-0.78247200
C	-3.25117500	-0.18336400	0.47389800
C	-4.07201000	0.28646700	1.50694400
H	-4.58515500	1.23038100	1.37268700
C	-4.21781800	-0.39395200	2.70543500
H	-4.83826400	0.01930400	3.49196500
C	-3.55146300	-1.59959400	2.89205900
H	-3.64372500	-2.14226300	3.82527100
C	-2.75212700	-2.09594600	1.87414700
H	-2.21666100	-3.02597800	2.01738400
C	-2.58125800	-1.41279400	0.66376600
C	-1.71039700	-2.03117200	-0.36512600
C	-0.65331500	-1.29247900	-0.94592500
C	0.17962500	-1.97826400	-1.83991000
H	1.01283600	-1.45297900	-2.28555700
C	-0.01915500	-3.31259300	-2.16899300
H	0.65591600	-3.80323400	-2.86092600
C	-1.07999400	-4.01620600	-1.60818500
H	-1.25216600	-5.05474500	-1.86504900
C	-1.91359000	-3.37235600	-0.70310900
H	-2.73821100	-3.91299400	-0.25284500
C	-1.78847000	1.62848800	-2.61377400
H	-2.54756600	2.40918400	-2.73508400
H	-0.82415700	2.13374800	-2.63056900
C	-1.85248000	0.67356800	-3.81351900
H	-1.11326100	-0.12123500	-3.70965300
H	-1.66077300	1.20251200	-4.75220500
H	-2.83214400	0.19738100	-3.88279600
C	-4.47029200	0.97683100	-1.41281800
H	-5.09521800	1.52223000	-0.69682900
H	-4.31068900	1.65397500	-2.25112600
C	-5.25214600	-0.25081400	-1.89845100
H	-4.66971600	-0.81042700	-2.63264500
H	-6.20251700	0.03676000	-2.35795500
H	-5.46633000	-0.92640900	-1.06843100

CAAC-Borepin Radical
E = -1636.7656454 a.u

C	-4.67500132	1.77583014	-0.26065361
C	-4.71862993	0.44033197	-0.55535239
C	-3.65011528	-0.52120215	-0.58021796
C	-3.54515073	2.58986084	0.09477074
C	-2.30101382	-0.35207057	-0.38874477
C	-2.21175802	2.26652722	0.14662433
H	-5.63525228	2.30339163	-0.28095749
H	-5.70913487	0.03278739	-0.78675436
H	-3.99140918	-1.54289418	-0.79256855
H	-3.81631728	3.62221025	0.35201038
H	-1.76559491	-1.29802029	-0.46912939
H	-1.60555439	3.11808963	0.46661637
B	-1.46912459	0.93071412	-0.12017413
C	0.07808435	0.93988034	-0.07414805
C	0.96676040	2.20301929	-0.17452476
N	0.91689299	-0.14665931	0.03905719
C	2.37023851	1.59973655	-0.42518646
C	2.36407630	0.20874372	0.20943709
H	2.50886722	1.47829353	-1.51097147
C	0.49285554	-1.51306517	0.09177760
C	0.06284098	-2.08756763	1.31111173
C	0.47599896	-2.26102732	-1.11229431

C	-0.32432002	-3.43602383	1.31111688
C	0.08025418	-3.60371242	-1.05927684
C	-0.30780074	-4.19408031	0.14281193
H	-0.66029769	-3.89475884	2.24391097
H	0.05910260	-4.19276328	-1.97878164
H	-0.61397481	-5.24325217	0.16487950
C	0.77719917	-1.63502558	-2.46957285
H	1.20489191	-0.64024765	-2.29127309
C	-0.51542350	-1.42348466	-3.27790132
H	-0.28451872	-0.96265098	-4.25248083
H	-1.02280646	-2.38346455	-3.46915958
H	-1.21886829	-0.76813324	-2.74598413
C	1.78907974	-2.45349669	-3.28558659
H	1.36392585	-3.41924756	-3.60301451
H	2.07316697	-1.90585389	-4.19874663
H	2.70501948	-2.66400635	-2.71304591
C	-0.07784598	-1.28979568	2.60110552
H	0.29686110	-0.27763483	2.41031820
C	0.73715645	-1.89860335	3.75345670
C	2.15412390	3.77296105	1.61044926
H	0.70481685	-1.23893550	4.63575112
H	0.32974872	-2.87661105	4.05681936
C	-1.55470795	-1.15122727	3.01130434
H	-1.99830184	-2.13385641	3.24187600
H	-1.64096480	-0.52436377	3.91411763
H	-2.15041781	-0.68759776	2.21290703
C	2.75648766	0.23998330	1.69669587
H	2.09716741	0.88787357	2.28666566
H	1.91761343	4.43268160	2.46131106
H	3.78680561	0.61517930	1.79436671
C	3.31366064	-0.75510025	-0.50505591
H	3.19441020	-1.78567026	-0.13947096
H	3.16942777	-0.74780280	-1.59153175
H	4.34986628	-0.44374325	-0.30345056
C	0.55187832	3.04230551	-1.41297883
C	1.42774548	4.25863229	-1.71715654
H	0.55368884	2.36446357	-2.28392478
C	0.88317370	3.05876119	1.13998444
H	0.10094980	3.81978637	0.99691102
H	0.51565293	2.42053555	1.95955833
H	3.19880653	2.21752124	-0.06073423
H	2.92667517	3.06855252	1.95191377
H	2.59979553	4.39703920	0.82206935
H	-0.48986189	3.37314121	-1.29621124
H	1.10346537	4.73803588	-2.65495420
H	2.48944409	3.98722819	-1.83570255
H	1.36312754	5.01958497	-0.92390084
H	2.72643697	-0.76917472	2.12815401
H	1.79183094	-2.04921099	3.47809114

CAAC-Borepin Anion
E = -1169.816224 a.u

C	-2.09855203	-4.58844908	-0.32571971
C	-0.75876924	-4.72913871	-0.51307487
C	0.29383144	-3.72133466	-0.45681347
C	-2.86979393	-3.38903033	-0.02810682
C	0.19138980	-2.36525103	-0.31584861
C	-2.45665480	-2.08503866	0.00736998
H	-2.69417943	-5.50949714	-0.38430811
H	-0.40145343	-5.74910140	-0.70616507
H	1.30337570	-4.14910700	-0.54805032
H	-3.92681201	-3.60038902	0.19221202
H	1.17655908	-1.89239478	-0.28952028
H	-3.27821899	-1.41724183	0.29632941
B	-1.05326857	-1.40301131	-0.20576999
C	-0.95279190	0.08526477	-0.23222024
C	-2.12464392	1.09324096	-0.36581708
N	0.24907778	0.89513435	-0.19409755
C	-1.40817164	2.40888840	-0.75702550
C	-0.00571414	2.34854501	-0.14793730
H	-1.29928778	2.42686632	-1.85358435
C	1.52343400	0.39703175	0.17542042
C	1.82737324	0.02466340	1.51450888
C	2.52130855	0.25087707	-0.82852169
C	3.13064940	-0.38614206	1.83046271
C	3.81286814	-0.15715587	-0.46554283
C	4.12755932	-0.46015113	0.85782510
H	3.36469854	-0.65877966	2.86316082
H	4.57928761	-0.26118429	-1.23866090
H	5.14024720	-0.77447916	1.12684375
C	2.19051425	0.41579167	-2.30725550
H	1.17571202	0.83110316	-2.35526399
C	2.15058736	-0.95237203	-3.01019300
H	1.86217262	-0.83385642	-4.06874200
H	3.13983309	-1.44122892	-2.98396449
H	1.42832602	-1.62574132	-2.52707089
C	3.15057858	1.36477831	-3.03746141
H	4.16684183	0.94059955	-3.10160911
H	2.80629669	1.54225058	-4.07031494
H	3.22699620	2.33894359	-2.53180451
C	0.77896119	-0.00007204	2.61912417
H	-0.15787408	0.36127919	2.18217108
C	1.15860038	0.89288080	3.81266152
C	-3.57779566	2.56311491	1.32868267
H	0.29108078	1.03257353	4.47948539
H	1.96392186	0.43774245	4.41375852
C	0.51221421	-1.43457668	3.10589880
H	1.41691908	-1.87698945	3.55709469
H	-0.27854177	-1.43407991	3.87581179
H	0.18420403	-2.07597610	2.27668647
C	0.02415512	2.93189286	1.28268558
H	-0.66961844	2.41779852	1.95741506
H	-4.19420305	2.46151404	2.23863073
H	-0.24456558	4.00104789	1.26258244
C	0.99090033	3.16776763	-0.98057758
H	2.02247176	3.03016692	-0.62110876
H	0.95523039	2.89162140	-2.04263063
H	0.74887854	4.24011371	-0.90148783
C	-3.06344857	0.69284689	-1.53837163
C	-4.21273037	1.64771481	-1.87154119
H	-2.42113006	0.57450604	-2.42745115
C	-2.92368936	1.22018477	0.97935551
H	-3.70018614	0.43808059	0.97868242
H	-2.24952555	0.94175284	1.80330756
H	-1.94652489	3.32260275	-0.47069361
H	-2.82937560	3.34437679	1.53376393
H	-4.23189115	2.94036991	0.52912477
H	-3.47662582	-0.30573070	-1.34589441
H	-4.74097870	1.31112890	-2.78000047
H	-3.86073443	2.67636877	-2.05943972
H	-4.96097916	1.69531381	-1.06442853
H	1.03537205	2.84862169	1.70704173
H	1.50406072	1.88751999	3.49382279

Cartesian coordinates optimized at the B3LYP-D3(BJ)/def2-TZVPD level

3b
E = -1636.653568 a.u

B	-0.61106100	-0.26581000	0.64291100
N	1.53908700	-0.43178100	-0.76625600
C	0.35037400	-0.99664300	-0.16110700
C	0.20458300	-2.44047200	-0.70370800
C	1.49230800	-2.63018800	-1.53030600
H	2.26366400	-3.09033300	-0.91008500
H	1.35777100	-3.27551100	-2.40025400
C	1.99266500	-1.22533300	-1.91841800
C	3.51200100	-1.22805100	-2.07669200
H	3.90028100	-0.22804600	-2.26840400
H	3.78965400	-1.86757400	-2.91806900
H	3.99995300	-1.60870200	-1.18031500
C	1.35683900	-0.75984700	-3.24646800
H	0.31122700	-0.49885400	-3.10006400
H	1.41409500	-1.55051700	-4.00012100
H	1.86928700	0.11480000	-3.64331800
C	0.10266000	-3.49572800	0.43584400

H	0.66419600	-3.12647200	1.29321400
H	-0.93838600	-3.53607900	0.76280900
C	0.58749800	-4.92040900	0.14168200
H	1.66264100	-4.94807700	-0.04515700
H	0.09634700	-5.36788100	-0.72113600
H	0.39481200	-5.56657800	1.00340100
C	-1.06956800	-2.51096500	-1.58617700
H	-1.90413100	-2.22013400	-0.95094500
H	-1.00545500	-1.73456600	-2.34588900
C	-1.41374400	-3.83664900	-2.26852700
H	-0.58382500	-4.23288800	-2.85990900
H	-2.25790400	-3.69266000	-2.94868000
H	-1.70712300	-4.60487900	-1.55101100
C	2.37777500	0.46135800	-0.07037500
C	3.02784200	0.07386500	1.12866000
C	3.84139500	0.98250400	1.79487700
H	4.33551100	0.67824100	2.71049900
C	4.05254200	2.26397600	1.30205900
H	4.69508900	2.95732100	1.83166100
C	3.42619100	2.64274400	0.12804900
H	3.56976900	3.64848100	-0.24840300
C	2.57961000	1.77388700	-0.55843200
C	1.84738800	2.28817500	-1.78174600
H	1.13413400	1.51562500	-2.05977400
C	2.79924700	2.53728900	-2.95906100
H	2.24038300	2.83284100	-3.85156600
H	3.38658800	1.65290400	-3.20511400
H	3.50055300	3.34214200	-2.72223700
C	1.04892700	3.56226900	-1.48498400
H	1.70625400	4.40357200	-1.25081400
H	0.37516500	3.41712500	-0.64629700
H	0.45352100	3.84153000	-2.35877900
C	2.88335600	-1.32111500	1.70171100
H	2.26196600	-1.88066300	1.01273700
C	2.15864000	-1.30353900	3.05081800
H	2.73028200	-0.75166700	3.80178100
H	2.01060800	-2.32134300	3.42248800
H	1.18066300	-0.83756800	2.94261700
C	4.23106900	-2.04376800	1.81667100
H	4.89011400	-1.55729700	2.53970000
H	4.75240400	-2.06637200	0.85822200
H	4.07970600	-3.07544800	2.14613500
C	-1.93156900	-0.88915500	1.29608100
C	-3.14495300	-0.60089900	0.78104000
C	-3.25009600	0.18432200	-0.47613400
C	-4.07078500	-0.28591100	-1.50928800
H	-4.58559000	-1.22886000	-1.37456800
C	-4.21534000	0.39378900	-2.70856000
H	-4.83562600	-0.01949700	-3.49520700
C	-3.54775200	1.59902600	-2.89584400
H	-3.63943000	2.14105800	-3.82948600
C	-2.74887300	2.09591500	-1.87757600
H	-2.21312900	3.02569200	-2.02131300
C	-2.57906300	1.41318800	-0.66668000
C	-1.70935500	2.03200300	0.36300100
C	-0.65270700	1.29367000	0.94521800
C	0.17858600	1.97957200	1.84081000
H	1.01061200	1.45424700	2.28865700
C	-0.02177600	3.31361400	2.17051200
H	0.65099300	3.80413600	2.86485500
C	-1.08224800	4.01703300	1.60806000
H	-1.25640600	5.05491300	1.86636200
C	-1.91373700	3.37313700	0.70076700
H	-2.73841400	3.91331700	0.24999900
C	-1.79050500	-1.62447500	2.61476000
H	-2.55069600	-2.40403900	2.73680900
H	-0.82685200	-2.13096700	2.63291400
C	-1.85403700	-0.66748700	3.81296700
H	-1.11311900	0.12570800	3.70877300
H	-1.66446500	-1.19525400	4.75275100
H	-2.83286500	-0.18929700	3.88030400
C	-4.47095100	-0.97368800	1.41082000
H	-5.09577300	-1.51914800	0.69476500
H	-4.31230600	-1.65024500	2.24977800
C	-5.25219900	0.25496400	1.89511200
H	-4.66999200	0.81462200	2.62947300
H	-6.20308300	-0.03157500	2.35419700
H	-5.46540900	0.93035200	1.06463900

Population Analysis

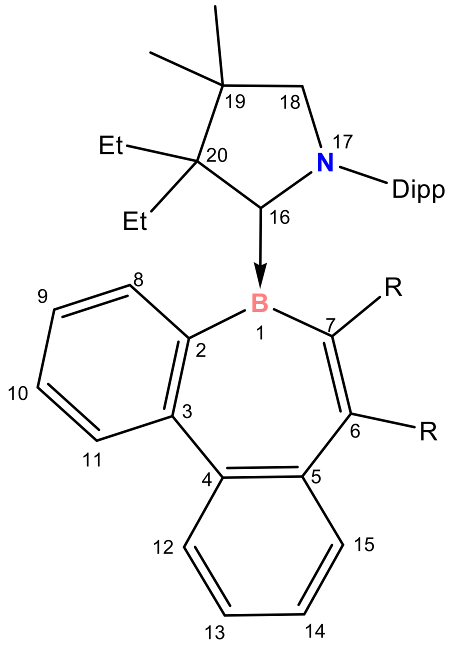

Table S3. Spin density comparison of **2a** (R=Ph) and **2b** (R=Et) at the B3LYP-D3(BJ)/def2-TZVP.

	**2a**	**2b**
B(1)	+0.315	+0.306
C(2)	-0.023	-0.022
C(3)	+0.014	+0.013
C(4)	-0.001	+0.000
C(5)	+0.002	+0.002
C(6)	+0.008	+0.013
C(7)	- 0.013	-0.018
C(8)	+0.016	+0.016
C(9)	-0.006	-0.006
C(10)	+0.010	+0.011
C(11)	-0.006	-0.007
C(12)	+0.002	+0.002
C(13)	+0.000	+0.000
C(14)	+0.001	+0.001
C(15)	+0.000	+0.000
C(16)	+0.421	+0.437
N(17)	+0.242	+0.237
C(18)	-0.015	-0.015
C(19)	+0.006	+0.006
C(20)	-0.032	-0.034

Molecular Orbitals

2a				
	Alpha		Beta	
LUMO+1	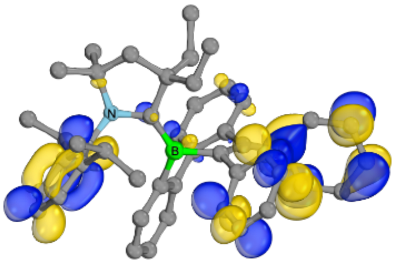		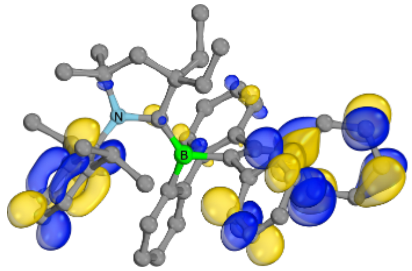	LUMO+2
LUMO	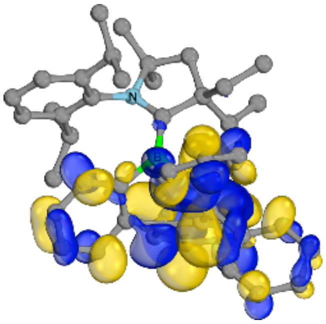		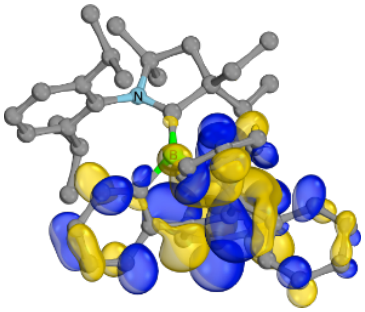	LUMO+1
SOMO	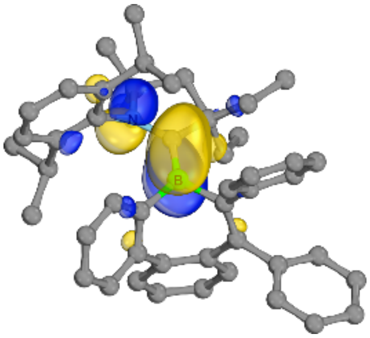		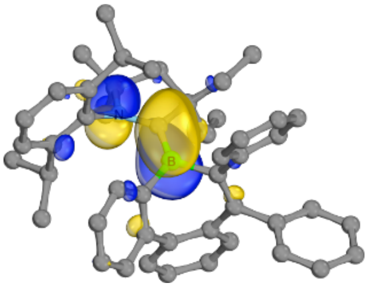	LUMO
HOMO-1	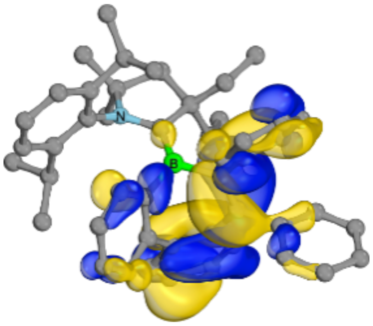		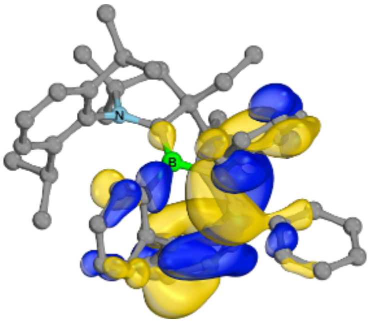	HOMO

Figure S11. Alpha and beta frontier molecular orbitals of **2a** computed at the UB3LYP-D3(BJ)/def2-TZVP level of theory.

2b				
	Alpha		Beta	
LUMO+1	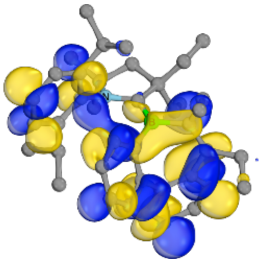		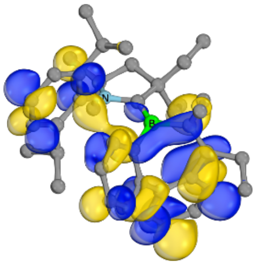	LUMO+2
LUMO	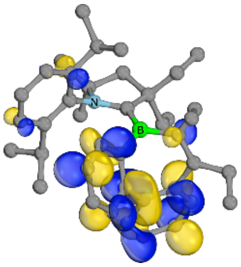		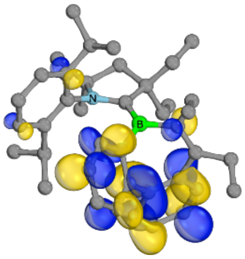	LUMO+1
SOMO	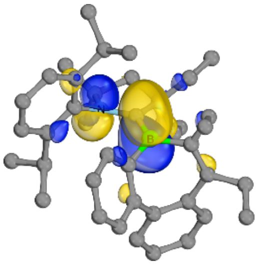		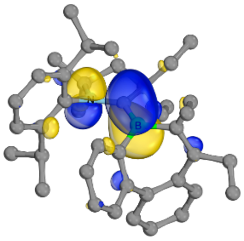	LUMO
HOMO-1	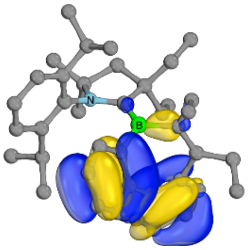		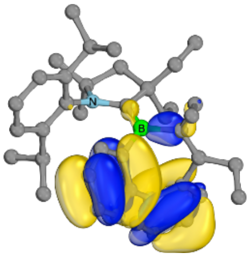	HOMO

Figure S12. Alpha and beta frontier molecular orbitals of **2b** computed at the uB3LYP-D3(BJ)/def2-TZVP level of theory.

TD-DFT (UV-Vis absorbance)

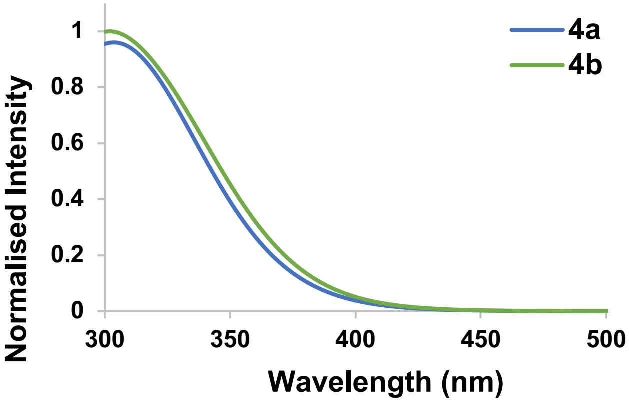

Figure S13. TD-DFT calculated absorbance spectrum of **2a** and **2b** at the ωB97XD/def2-SVP//B3LYP-D3(BJ)/def2-TZVP (SMD, Toluene) level of theory.

Comparison of Experimental and Theoretical Geometries

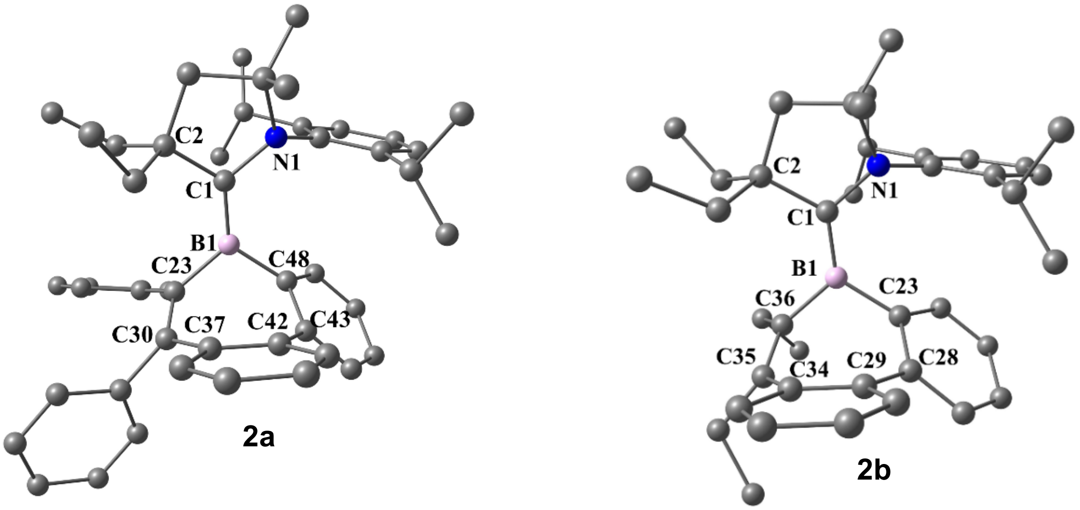

2a			2b		
Parameters	Expt (Å or °)	Calc (Å or °)	Parameters	Expt (Å or °)	Calc (Å or °)
C1-B1	1.517(5)	1.511	C1-B1	1.544(2)	1.512
C1-N1	1.385(4)	1.370	C1-N1	1.366(2)	1.371
C1-C2	1.546(4)	1.538	C1-C2	1.543(2)	1.539
B1-C48	1.574(5)	1.573	B1-C36	1.594(2)	1.581
B1-C23	1.596(5)	1.590	B1-C23	1.591(2)	1.575
C23-C30	1.352(5)	1.348	C36-C35	1.356(2)	1.347
C48-C43	1.406(5)	1.411	C35-C34	1.489(2)	1.485
C43-C42	1.503(5)	1.485	C34-C29	1.412(2)	1.413
C42-C37	1.425(5)	1.414	C29-C28	1.493(2)	1.484
C30-C37	1.495(5)	1.483	C28-C23	1.423(2)	1.412
C2-C1-N1	108.2(3)	108.6	C2-C1-N1	107.6(1)	108.6
C2-C1-B1	125.3(3)	125.6	C2-C1-B1	123.9(1)	125.1
N1-C1-B1	125.7(3)	125.4	N1-C1-B1	128.5(1)	125.8
C1-B1-C23	122.7(3)	124.6	C1-B1-C36	121.5(1)	124.2
C1-B1-C48	127.6(3)	127.1	C1-B1-C23	128.9(1)	127.5
C23-B1-C48	109.2(3)	107.5	C23-B1-C36	109.4(1)	107.9

Figure S14. The experimental and theoretical geometrical parameters of **2a**, and **2b** complexes.

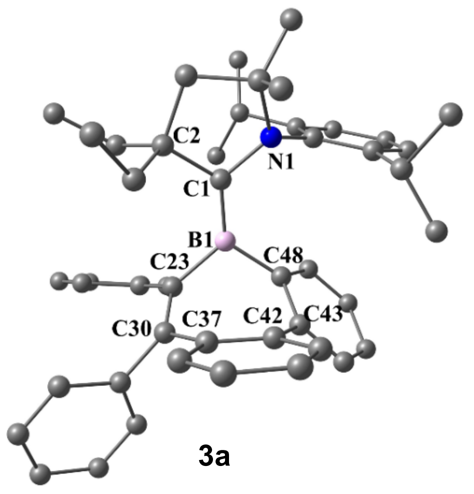

3a

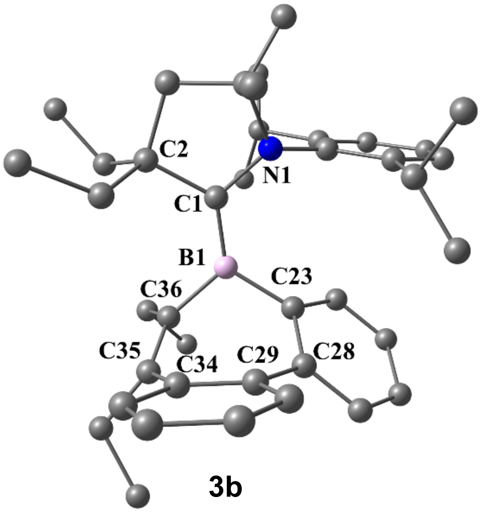

3b

3a			3b		
Parameters	Expt (Å or °)	Calc (Å or °)	Parameters	Expt (Å or °)	Calc (Å or °)
C1-B1	1.443(12)	1.449	C1-B1	1.471(11)	1.451
C1-N1	1.463(10)	1.445	C1-N1	1.484(10)	1.449
C1-C2	1.551(11)	1.549	C1-C2	1.557(11)	1.549
B1-C48	1.609(12)	1.586	B1-C36	1.617(13)	1.600
B1-C23	1.620(12)	1.607	B1-C23	1.585(12)	1.589
C23-C30	1.346(10)	1.350	C36-C35	1.349(12)	1.349
C48-C43	1.436(10)	1.414	C35-C34	1.478(11)	1.486
C43-C42	1.486(10)	1.484	C34-C29	1.445(13)	1.413
C42-C37	1.407(12)	1.413	C29-C28	1.465(13)	1.483
C30-C37	1.492(12)	1.484	C28-C23	1.425(11)	1.414
C2-C1-N1	105.9(6)	107.0	C2-C1-N1	106.2(6)	107.1
C2-C1-B1	128.1(7)	126.8	C2-C1-B1	126.0(8)	127.0
N1-C1-B1	125.4(7)	125.5	N1-C1-B1	127.5(7)	125.4
C1-B1-C23	124.1(7)	125.2	C1-B1-C36	125.3(8)	125.3
C1-B1-C48	128.5(8)	128.3	C1-B1-C23	127.3(8)	128.1
C23-B1-C48	107.2(6)	106.1	C23-B1-C36	107.4(7)	106.5

Figure S15. The experimental and theoretical geometrical parameters of **3a** and **3b** complexes.

3a	
LUMO+1	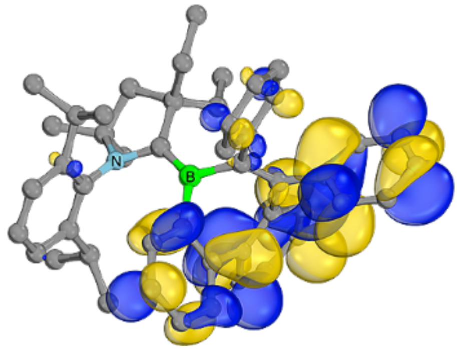
LUMO	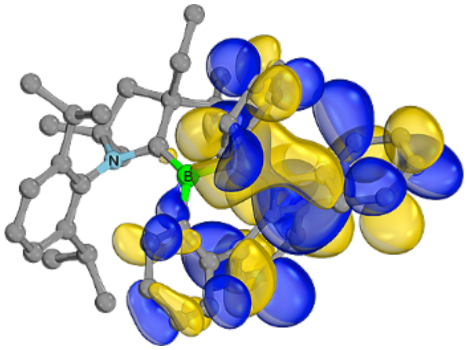
HOMO	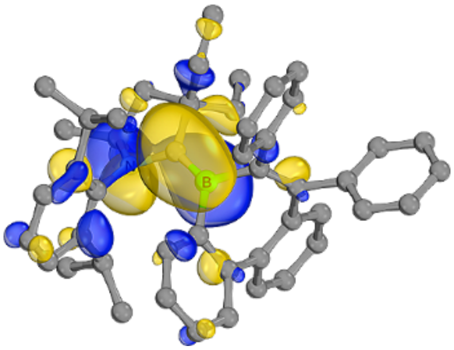
HOMO-1	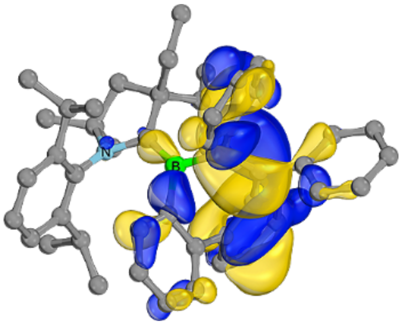

Figure S16. Frontier molecular orbitals of **3a** computed at the B3LYP-D3(BJ)/def2-TZVP level of theory.

3b	
**LUMO+1**	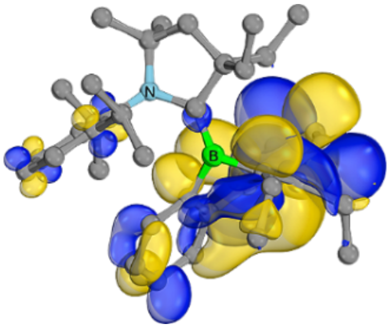
**LUMO**	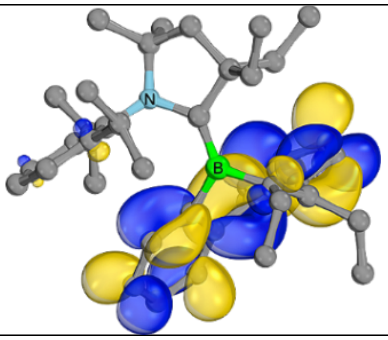
**HOMO**	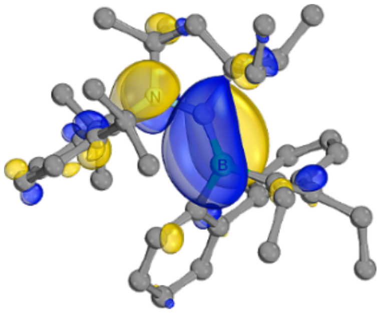
**HOMO-1**	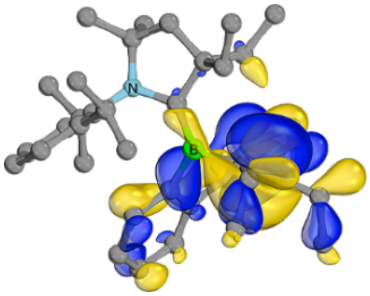

Figure S17. Frontier molecular orbitals of **3b** computed at the B3LYP-D3(BJ)/def2-TZVP level of theory.

EDA-NOCV analysis

2a	
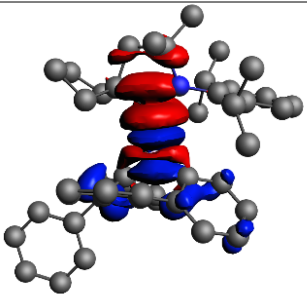<br>$\Delta E_{orb(1)} = -124.2, \lvert v \rvert = 0.71$	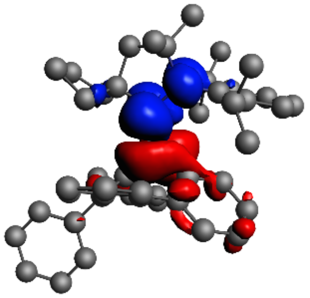<br>$\Delta E_{orb(2)} = -49.4, \lvert v \rvert = 0.79$
**2b**	
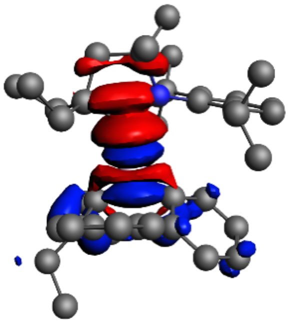<br>$\Delta E_{orb(1)} = -119.1, \lvert v \rvert = 0.70$	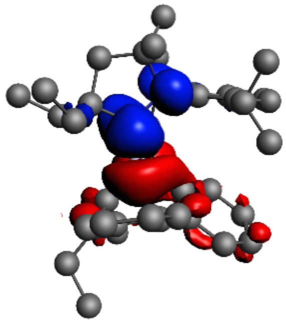<br>$\Delta E_{orb(2)} = -49.1, \lvert v \rvert = 0.79$
**3a**	
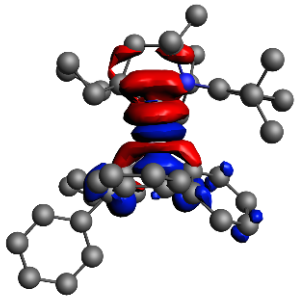<br>$\Delta E_{orb(1)} = -132.4, \lvert v \rvert = 0.80$	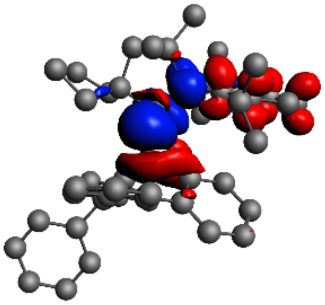<br>$\Delta E_{orb(2)} = -85.4, \lvert v \rvert = 1.35$
**3b**	
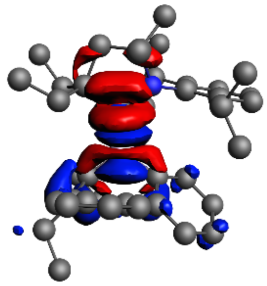<br>$\Delta E_{orb(1)} = -133.1, \lvert v \rvert = 0.78$	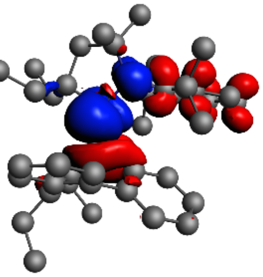<br>$\Delta E_{orb(2)} = -83.8, \lvert v \rvert = 1.35$

Figure S18. Shape of the deformation densities $\Delta\rho_{(1)}$ and $\Delta\rho_{(2)}$, which are associated with the orbital interactions $\Delta E_{orb(1)}$ and $\Delta E_{orb(2)}$ in **2a**, **2b, 3a** and **3b**, and eigenvalues $|v|$ of the charge flow. The isosurface value is 0.001 au. The color code of the charge flow is red → blue. $|v_n|$ gives the size of the charge transfer.

Table S4. EDA-NOCV results of **2a** at the B3LYP-D3(BJ)/TZ2P//B3LYP-D3(BJ)/def2-TZVP level. The interacting fragments are CAAC and dibenzoborepin, with different charge and multiplicity labeled as [S] singlet, [D] doublet, [T] triplet. Energy values are given in kcal/mol.

Energy terms	Donor-Acceptor [Dibenzoborepin] [D] + [CAAC] [S]	Electron-sharing + donor-acceptor [Dibenzoborepin] [D] + [CAAC] [T]	Donor-Acceptor $[Dibenzoborepin]^+$ [S] + $[CAAC]^-$ [D]	Electron-sharing + donor-acceptor $[Dibenzoborepin]^-$ [T] + $[CAAC]^+$ [D]
ΔE_{int}	-155.1	-174.7	-286.1	-271.2
ΔE_{Pauli}	265.2	292.7	349.5	352.3
ΔE_{disp}[a]	-29.7 (7.1%)	-29.7 (6.4%)	-29.7 (4.7%)	-29.7 (4.8%)
ΔE_{elstat}[a]	-184.6 (43.9%)	-198.6 (42.5%)	-290.4 (45.7%)	-269.3 (43.2%)
ΔE_{orb}[a]	-206.1 (49.0%)	-239.0 (51.1%)	-315.5 (49.6%)	-324.4 (52.0%)

[a] The values in parentheses give the percentage contribution to the total attractive interactions ΔE_{elstat} + ΔE_{orb} + ΔE_{disp}.

Table S5. EDA-NOCV results of **2b** at the B3LYP-D3(BJ)/TZ2P//B3LYP-D3(BJ)/def2-TZVP level. The interacting fragments are CAAC and diethyl substituted dibenzoborepin, with different charge and multiplicity labeled as [S] singlet, [D] doublet, [T] triplet. Energy values are given in kcal/mol.

Energy terms	Donor-Acceptor [Diethyl substituted dibenzoborepin] [D] + [CAAC] [S]	Electron-sharing + donor-acceptor [Diethyl substituted dibenzoborepin] [D] + [CAAC] [T]	Donor-Acceptor [Diethyl substituted $dibenzoborepin]^+$ [S] + $[CAAC]^-$ [D]	Electron-sharing + donor-acceptor [Diethyl substituted $dibenzoborepin]^-$ [T] + $[CAAC]^+$ [D]
ΔE_{int}	-161.1	-181.3	-288.4	-273.3
ΔE_{Pauli}	263.9	288.3	338.8	359.9
ΔE_{disp}[a]	-29.8 (7.0%)	-29.8 (6.3%)	-29.8 (4.8%)	-29.8 (4.7%)
ΔE_{elstat}[a]	-184.9 (43.5%)	-199.4 (42.5%)	-288.0 (45.9%)	-283.7 (44.0%)
ΔE_{orb}[a]	-210.2 (49.5%)	-240.4 (51.2%)	-309.3 (49.3%)	-319.7 (50.5%)

[a] The values in parentheses give the percentage contribution to the total attractive interactions ΔE_{elstat} + ΔE_{orb} + ΔE_{disp}.

Table S6. EDA-NOCV results of **3a** at the B3LYP-D3(BJ)/TZ2P//B3LYP-D3(BJ)/def2-TZVP level. The interacting fragments are CAAC and dibenzoborepin, with different charge and multiplicity labeled as [S] singlet, [D] doublet, [T] triplet. Energy values are given in kcal/mol.

Energy terms	Electron-sharing + donor-acceptor [Dibenzoborepin] [D] + $[CAAC]^-$ [D]	Donor-Acceptor $[Dibenzoborepin]^-$ [S] + [CAAC] [S]	Electron-sharing $[Dibenzoborepin]^-$ [T] + [CAAC] [T]	Donor-Acceptor $[Dibenzoborepin]^+$ [S] + $[CAAC]^{2-}$ [S]
ΔE_{int}	-202.6	-199.8	-187.7	-411.4
ΔE_{Pauli}	295.9	276.2	330.6	396.2
ΔE_{disp}[a]	-28.3 (5.7%)	-28.3 (5.9%)	-28.3 (5.5%)	-28.3 (3.5%)
ΔE_{elstat}[a]	-212.5 (42.6%)	-187.4 (39.4%)	-219.3 (42.3%)	-370.5 (45.9%)
ΔE_{orb}[a]	-257.6 (51.7%)	-260.3 (54.7%)	-270.6 (52.2%)	-408.8 (50.6%)

[a] The values in parentheses give the percentage contribution to the total attractive interactions ΔE_{elstat} + ΔE_{orb} + ΔE_{disp}.

Table S7. EDA-NOCV results of **3b** at the B3LYP-D3(BJ)/TZ2P//B3LYP-D3(BJ)/def2-TZVP level. The interacting fragments are CAAC and diethyl substituted dibenzoborepin, with different charge and multiplicity labeled as [S] singlet, [D] doublet, [T] triplet. Energy values are given in kcal/mol.

Energy terms	Electron-sharing + donor-acceptor [Diethyl substituted dibenzoborepin] [D] + $[CAAC]^-$ [D]	Donor-Acceptor [Diethyl substituted $dibenzoborepin]^-$ [S] + [CAAC] [S]	Electron-sharing [Diethyl substituted $dibenzoborepin]^-$ [T] + [CAAC] [T]	Donor-Acceptor [Diethyl substituted $dibenzoborepin]^+$ [S] + $[CAAC]^{2-}$ [S]
ΔE_{int}	-195.5	-199.4	-186.1	-404.2
ΔE_{Pauli}	296.6	286.6	334.4	378.8
ΔE_{disp}[a]	-25.7 (5.2%)	-25.7 (5.3%)	-25.7 (4.9%)	-25.7 (3.3%)
ΔE_{elstat}[a]	-211.7 (43.0%)	-194.5 (40.0%)	-221.7 (42.6%)	-363.8 (47.1%)
ΔE_{orb}[a]	-254.8 (51.8%)	-265.8 (54.7%)	-273.1 (52.5%)	-383.6 (49.6%)

[a] The values in parentheses give the percentage contribution to the total attractive interactions ΔE_{elstat} + ΔE_{orb} + ΔE_{disp}.

Table S8. EDA-NOCV results of **2a**, **2b** and their anionic analogues at the B3LYP-D3(BJ)/TZ2P//B3LYP-D3(BJ)/def2-TZVP level. The interacting fragments are singlet CAAC and doublet dibenzoborepin or diethyl substituted dibenzoborepin for **2a** and **2b**, and doublet $[CAAC]^-$ and doublet dibenzoborepin or diethyl substituted dibenzoborepin for **3a** and **3b**. Energy values are given in kcal/mol.

Energy terms	Interaction	**2a**	**2b**	**3a**	**3b**
ΔE_{int}		-155.1	-161.1	-202.6	-195.5
ΔE_{Pauli}		265.2	263.9	295.9	296.6
ΔE_{disp}[a]		-29.7 (7.1%)	-29.8 (7.0%)	-28.3 (5.7%)	-25.7 (5.2%)
ΔE_{elstat}[a]		-184.6 (43.9%)	-184.9 (43.5%)	-212.5 (42.6%)	-211.7 (43.0%)
ΔE_{orb}[a]		-206.1 (49.0%)	-210.2 (49.5%)	-257.6 (51.7%)	-254.8 (51.8%)
$\Delta E_{orb(1)}$[b]	$[Borepin]\leftarrow[CAAC]^q$ σ-donation	-124.2 (60.3%)	-119.1 (56.7%)	-132.4 (51.4%)	-133.1 (52.2%)
$\Delta E_{orb(2)}$[b]	[Borepin]→[CAAC] π-backdonation	-49.4 (24.0%)	-49.1 (23.4%)		
	$[Borepin]–[CAAC]^-$ electron-sharing π-bond			-85.4 (33.2%)	-83.8 (32.9%)
$\Delta E_{orb(rest)}$[b]		-32.5 (15.8%)	-42.0 (20.0%)	-39.8 (15.5%)	-37.9 (14.9%)

[a] The values in parentheses give the percentage contribution to the total attractive interactions $\Delta E_{elstat} + \Delta E_{orb} + \Delta E_{disp}$. [b] The values in parentheses give the percentage contribution to the total orbital interactions ΔE_{orb}.

CAAC-Borepin Radical	CAAC-Borepin Anion
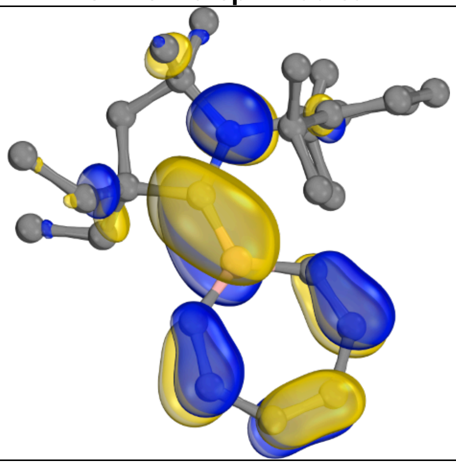	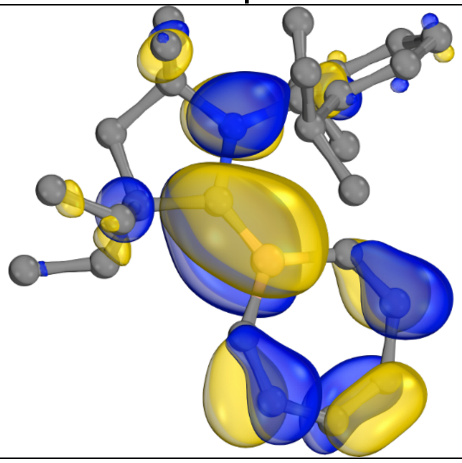
**Alpha SOMO**	**HOMO**
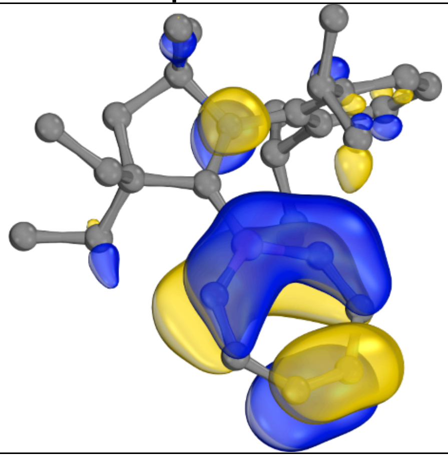	
**Beta SOMO**	

Figure S19. SOMO and HOMO molecular orbitals of radical and anion unsubstituted CAAC-borepin respectively (B3LYP-D3(BJ)/def2-TZVP).

References

[1] A. J. Arduengo, R. Krafczyk, R. Schmutzler, H. A. Craig, J. R. Goerlich, W. J. Marshall, M. Unverzagt, *Tetrahedron* **1999**, *55*, 14523-14534.

[2] R. Jazzar, R. D. Dewhurst, J.-B. Bourg, B. Donnadieu, Y. Canac, G. Bertrand, *Angew. Chem. Int. Ed.* **2007**, *46*, 2899-2902.

[3]

[4] G. Sheldrick, *Acta Cryst. A* **2015**, *71*, 3-8.

[5] O. V. Dolomanov, L. J. Bourhis, R. J. Gildea, J. A. K. Howard, H. Puschmann, *J. Appl. Crystallogr.* **2009**, *42*, 339-341.

[6] A. Spek, *Acta Cryst. C* **2015**, *71*, 9-18.

[7] G. Sheldrick.

[8] a) A. D. Becke, *J. Chem. Phys.* **1993**, *98*, 1372-1377; b) A. D. Becke, *J. Chem. Phys.* **1993**, *98*, 5648-5652; c) C. Lee, W. Yang, R. G. Parr, *Phys. Rev. B* **1988**, *37*, 785-789; d) S. Grimme, J. Antony, S. Ehrlich, H. Krieg, *J. Chem. Phys.* **2010**, *132*, 154104; e) S. Grimme, S. Ehrlich, L. Goerigk, *J. Comput. Chem.* **2011**, *32*, 1456-1465.

[9] F. Weigend, R. Ahlrichs, *Phys. Chem. Chem. Phys.* **2005**, *7*, 3297-3305.

[10] D. P. Chong, in *Recent Advances in Density Functional Methods*, pp. i-v.

[11] F. Neese, *WIREs Comput Mol Sci* **2012**, *2*, 73-78.

[12] a) S. Stoll, A. Schweiger, *J. Magn. Reson.* **2006**, *178*, 42-55; b) S. Stoll, R. D. Britt, *Phys. Chem. Chem. Phys.* **2009**, *11*, 6614-6625; c) S. Stoll, in *Multifrequency Electron Paramagnetic Resonance: Data and Techniques* (Ed.: S. K. Misra), Wiley-VCH, **2014**, pp. 69-138.

[13] A. V. Marenich, S. V. Jerome, C. J. Cramer, D. G. Truhlar, *J. Chem. Theory Comput* **2012**, *8*, 527-541.

[14] G. te Velde, F. M. Bickelhaupt, E. J. Baerends, C. Fonseca Guerra, S. J. A. van Gisbergen, J. G. Snijders, T. Ziegler, *J. Comput. Chem.* **2001**, *22*, 931-967.

[15] a) L. Zhao, M. Hermann, N. Holzmann, G. Frenking, *Coord. Chem. Rev.* **2017**, *344*, 163-204; b) G. Frenking, M. Hermann, D. M. Andrada, N. Holzmann, *Chem. Soc. Rev.* **2016**, *45*, 1129-1144; c) L. Zhao, S. Pan, N. Holzmann, P. Schwerdtfeger, G. Frenking, *Chem. Rev.* **2019**; d) G. Frenking, F. M. Bickelhaupt, in *The Chemical Bond: Fundamental Aspects of Chemical Bonding* (Eds.: G. Frenking, S. Shaik), Eds. Wiley-VCH: Weinheim, **2014**, pp. 121-157.
